# Selective Removal of Sodium Salt Taste Disrupts the Maintenance of Dendritic Architecture of Gustatory Relay Neurons in the Mouse Nucleus of the Solitary Tract

**DOI:** 10.1523/ENEURO.0140-20.2020

**Published:** 2020-10-19

**Authors:** Rolf Skyberg, Chengsan Sun, David L. Hill

**Affiliations:** Department of Psychology, University of Virginia, Charlottesville, VA 22904-4400

**Keywords:** activity dependent, dendritic maintenance, epithelial sodium channel, gustatory circuit, nucleus of the solitary tract, taste

## Abstract

Neuronal activity plays critical roles in the development of sensory circuits in the mammalian brain. Experimental procedures are now available to alter the function of specific taste transduction pathways and have been especially useful in studying how stimulus-specific taste activity influences the development of central gustatory circuits. We previously used a mouse knock-out (KO) model in which the transduction channel necessary for sodium taste is removed from taste bud cells throughout life. In these KO mice, the terminal fields that carry taste information from taste buds into the nucleus of the solitary tract (NST) fail to mature, suggesting that sodium-elicited taste activity is important for the proper development of central gustatory circuits. Here, we tested the hypothesis that the development and maintenance of the dendritic architecture of NST relay cells, the primary postsynaptic partner of gustatory nerve terminal fields, are similarly dependent on sodium-elicited taste activity. The dendritic fields of NST relay cells, from adult male and female mice in which the α-subunit of the epithelial sodium channel (αENaC) was conditionally deleted in taste bud cells throughout life, were up to 2.4× larger and more complex than that of age-matched control mice. Interestingly, these differences in dendritic architecture did not appear until after the age when terminal fields begin “pruning,” after postnatal day (P)20. Overall, our results suggest that ENaC-mediated sodium taste activity is necessary for the maintenance of dendritic fields of relay cells in the gustatory NST.

## Significance Statement

Neural activity plays major roles in the development of sensory circuits in the mammalian brain. Here, we tested whether loss of sodium taste activity throughout development impacts the dendritic development of cells that relay peripheral taste information to more central structures, in the nucleus of the solitary tract (NST). We found that the dendritic fields of NST relay neurons in mice without sodium taste activity throughout development increased in size at about the age that normal “pruning” occurs in gustatory nerve terminal fields. Our findings suggest a novel role for sodium taste activity in the maintenance of NST relay cell dendritic architecture and highlight a level of plasticity not seen in other sensory systems.

## Introduction

Neuronal activity plays important developmental roles in both subcortical and cortical sensory circuits (Brunjes, 1994; Cang et al., 2005; Lee, 2005; Leake et al., 2006; Guido, 2008; Sun et al., 2017). For instance, developing visual circuits in the dorsal lateral geniculate nucleus (dLGN) are shaped by both spontaneous and visually-evoked activity in the retina (Katz and Shatz, 1996; Hooks and Chen, 2006). Removal of this activity has several effects on developing dLGN circuits in preventing the pruning of retinal ganglion cell terminal fields (Katz and Shatz, 1996; Stellwagen and Shatz, 2002), inhibiting synaptic refinement (Hooks and Chen, 2006), and altering both dendritic morphology and physiology of postsynaptic thalamocortical relay cells (Wiesel and Hubel, 1963; Friedlander et al., 1982). Thus, neural activity regulates multiple aspects of the developing visual pathway even at the first synaptic relay in the brain.

By comparison, less is known about roles neural activity play in developing subcortical gustatory circuits, areas important in regulating feeding and motivated behaviors (Spector and Travers, 2005; Spector and Glendinning, 2009). Terminal fields of nerves that carry gustatory information from taste buds to the brain undergo substantial activity-dependent refinement throughout early life, similar to developing retinogeniculate terminal fields in the dLGN (Katz and Shatz, 1996; Sun et al., 2017). Initially, chorda tympani (CT), greater superficial petrosal (GSP), and glossopharyngeal (IX) terminal fields in the rodent nucleus of the solitary tract (NST) are large and overlap extensively (Mangold and Hill, 2008). Throughout postnatal development these terminal fields are “pruned” into a smaller, more segregated organization (Sollars et al., 2006; Mangold and Hill, 2008; Zheng et al., 2014). This anatomic development occurs in tandem with a nearly two-fold developmental increase in the magnitude of salty-taste responses (and to a lesser extent sweet-taste responses) in the CT (Yamada, 1980; Hill and Bour, 1985; Hill, 1988; Zheng et al., 2014), suggesting increases in peripheral input regulates terminal field “pruning.” Sun et al. (2017) confirmed this relationship by using mice in which the channel that transduces salty taste stimuli, the epithelial sodium channel (ENaC), was genetically deleted from taste bud cells throughout an animals’ life [ENaC knock-out (KO) mice; Chandrashekar et al., 2010; Sun et al., 2017]. These ENaC KO mice had significantly diminished CT and GSP nerve responses to NaCl, did not exhibit normal appetitive licking behaviors to NaCl, and had large, overlapping, unrefined CT and GSP terminal fields in adulthood (Chandrashekar et al., 2010; Sun et al., 2017). Interestingly, removal of ENaC activity in adulthood, after terminal fields reached maturity, reverts the terminal field organization to a large, overlapping, and immature state (Skyberg et al., 2017). Therefore, salt taste activity from a single transduction pathway is necessary for the proper development and for the maintenance of gustatory terminal fields in control mice.

Given the large effects that removing sodium salt taste has on gustatory terminal fields, one might expect removing ENaC-mediated neural activity would also affect their postsynaptic targets that send afferent taste information to more central structures. A few studies suggest a role for taste activity in the development of dendritic architecture of NST relay cells, the primary postsynaptic partners of gustatory terminal field; however, these studies have not shown this conclusively (Lasiter and Kachele, 1990; Lasiter, 1991). To test this hypothesis directly, we examined the development of dendritic size, shape, and spread in postnatal day (P)20 and adult control and ENaC KO male and female mice. Our results show that NST relay cell dendritic architecture in normally-developing and ENaC KO mice reaches maturity by P20. This age is before sodium salt taste activity fully matures in controls. Unexpectedly, we found that the size of dendritic fields in ENaC KO mice became larger and more complex than controls with age, while the general shape of the dendritic fields remained similar to controls. Our surprising findings indicate that, while ENaC-mediated sodium salt taste activity appears not to be involved in the normal dendritic development of NST relay cells in control mice, it is required to maintain their mature dendritic organization.

## Materials and Methods

### Animals

All experiments were approved by the University of Virginia Animal Care and Use Committee and followed guidelines set forth by the National Institutes of Health and the Society for Neurosciences. To examine the role of sodium salt taste on the development and organization of gustatory relay cell dendrites in the rostral NST, we used mice described in detail by Chandrashekar et al. (2010). Briefly, the α-subunit of the ENaC (αENaC) was conditionally deleted in taste bud cells by crossing mice that drove the expression of Cre-recombinase under the cytokeratin 19 (CreK19) promotor with mice that were homozygous for the floxed *Scnn1a* (αENaC) gene (*Scnn1a^flox/flox^*). The CreK19 mice were generously supplied by Charles Zuker and Edith Hummler supplied the *Scnn1a^flox/flox^*mice. Therefore, our experimental animals had the genotype K19-Cre *Scnn1a^flox/flox^* (αENaC KO). The control group consisted of mice that were littermates to experimental animals, but did not have the CreK19 promoter (*Scnn1a^flox/flox^*). To investigate the morphologic development of dendrites, we retrogradely labeled relay cells in the NST at two significant developmental ages. First, we labeled NST relay cells in control and αENaC KO mice between P18 and P22 (P20 controls, four mice, two male/two female, 22 cells; P20 αENaC KO, three mice, two male/one female, 31 cells). We chose this age group for three reasons. At this age taste responses to NaCl have not fully matured (Hill and Bour, 1985; Zheng et al., 2014), allowing us to assess how dendritic morphologies are organized before peripheral taste input to the NST has matured. The terminal fields of gustatory nerves in the NST begin their developmental pruning around this time, suggesting this is a period when substantial functional and structural reorganization within the NST occurs (Sollars et al., 2006; Mangold and Hill, 2008; Zheng et al., 2014). And finally, animals younger than P18 were too small to reliably fit into the stereotaxic device, making consistent targeting of the parabrachial nucleus (PBN; the next central relay beyond the NST) difficult. We also labeled NST relay cells in control and αENaC KO mice between 60 and 120 d of age (adult control, eight mice, three male/five female, 123 cells; adult αENaC KO, 10 mice, four male/six female, 81 cells). At these ages, both the peripheral taste responses and gustatory terminal fields in the NST are fully matured in control mice (Sollars et al., 2006; Mangold and Hill, 2008; Zheng et al., 2014; Sun et al., 2017). There were no sex-related differences found in any of our experiments or analyses; therefore, male and female mice within the same experimental group were combined.

### PBN injections

**Figure 1.**
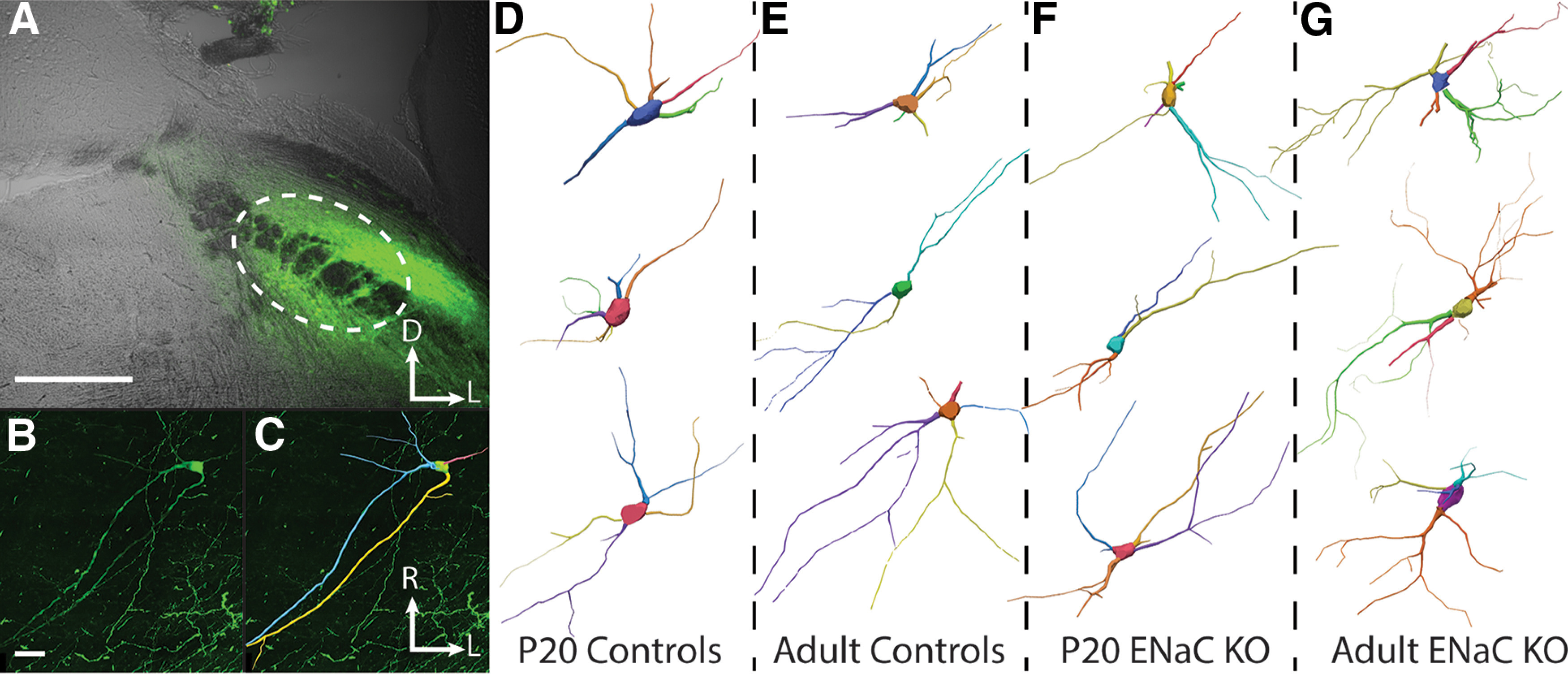
Retrograde labeling and tracing of NST relay cells. ***A-C***, Stereotaxic injections into the waist region of the PBN results in robust retrograde labeling of relay cells in the NST. ***A***, Coronal section through the PBN showing a stereotaxically-guided injection of 10% BDA (green) into the waist region of the right PBN (outlined with white dotted line). Photomicrograph taken at 4x. Scale bar in A, 500mm. D, dorsal; L, lateral. ***B***, Horizontal section through the right NST showing a retrogradely-labeled NST relay cell following an injection of 10% BDA into the right PBN. Photomicrograph taken at 60x. ***C***, Tracing of the same NST relay cell shown in B. R, rostral; L, lateral. ***D-G***, Representative NST relay cell tracings from P20 control (***D***), adult control (***E***), P20 ENaC KO (***F***), and adult ENaC KO mice (***G***). Scale bar in ***B***, 20mm and also applies to ***C-G***.

To selectively label NST relay cells, we injected the retrograde tracer biotinylated dextran amine (BDA; Invitrogen; D7135) into the “waist” region of the PBN (Fig. 1*A*). This area is the primary target for NST relay cell axons and retrograde tracer injections here result in Golgi-like labeling of relay cells in the NST (Fig. 1*B–G*; Norgren and Leonard, 1971; Norgren and Pfaffmann, 1975; Whitehead, 1990; Tokita et al., 2009; Tokita and Boughter, 2016). Mice were sedated with a 0.32 mg/kg (0.24 mg/kg for P20 mice) injection of Domitor (medetomidine hydrochloride, i.m.; Pfizer Animal Health) and anesthetized with 40 mg/kg (30 mg/kg for P20 mice) Ketaset (ketamine hydrochloride, i.m.; Fort Dodge Animal Health). A water-circulating heating pad was used to maintain body temperature. Mice were placed in a non-traumatic head holder (Erickson, 1966), and an incision was made along the midline on the top of the head to expose the animal’s skull. Bregma and λ were aligned horizontally and two 2 × 2-mm bilateral holes were drilled in the skull ∼5.2 mm (5.1 mm for P20 mice) caudal to bregma and 1.2 mm (1.1 mm for P20 mice) lateral to the midline (similar to Tokita et al., 2009; Tokita and Boughter, 2016). A glass pipette filled with 10% 3 kDa BDA in 0.1 m citrate/NaOH buffer (pH 3.0) was slowly lowered 2.8 mm (2.7 mm for P20 mice) from the top of the cerebellum. This acidic solvent increased retrograde axonal uptake of the tracer and provided Golgi-like cell labeling in the NST (Fig. 1; Kaneko et al., 1996; Reiner et al., 2000; Corson and Erisir, 2013). BDA was injected iontophoretically with a 6-μA positive current (7 s on, 7 s off) for 10 min (Reiner et al., 2000; Fekete et al., 2015). Following the injection, the pipette tip was left in place without any positive current for 5 min to allow time for tracer uptake and to reduce backflow through the injection site. The pipette was then slowly raised out of the brain and the procedure was repeated on the other side of the brain. The animal’s scalp was then sutured or sealed using Vetbond and the animal was injected with 5 mg/ml (3.75 mg/ml for P20 mice) Antisedan (atipamezole hydrochloride, i.m.; Pfizer Animal Health) to promote reversal of anesthesia.

### CT nerve label

Procedures used to label the CT terminal field with fluorescent tracers were similar to that described previously in mouse (Sun et al., 2015, 2017; Skyberg et al., 2017). Briefly, 24 h after PBN injections, animals were anesthetized as described above, placed into a non-traumatic head holder (Erickson, 1966) and maintained at 36°C with a water-circulating heating pad. The animal was placed into the supine position and an incision was made along the ventral midline of the neck. Musculature was retracted to expose the right tympanic bulla. The bulla was opened and the CT was cut peripheral to the geniculate ganglion. Crystals of 3-kDa tetramethyrhodamine dextran amine (TMR; Thermofisher Scientific; D3308, RRID: AB_2315472) were placed on the central stump of the CT, and the opening of the bulla was filled with Kwik-Sil (World Precision Instruments) to prevent crystals from diffusing from the site of the intended label. The incision was then sutured and the animal was revived using Antisedan (atipamezole hydrochloride, i.m.; Pfizer Animal Health).

### Tissue preparation

Following a 48-h survival period, animals were deeply anesthetized with urethane and transcardially perfused with Krebs–Henseleit buffer (pH 7.3), followed by 4% paraformaldehyde (pH 7.2). Brains were removed, postfixed in 4% paraformaldehyde overnight, and sectioned horizontally on a vibratome at 100 μm. We chose to section tissue in the horizontal plane, as the dendritic arbors run parallel to this plane of sectioning, thus reducing the number of transected dendrites. It is also the plane in which the gustatory nerve axons branch from the solitary tract and project medially (Davis and Jang, 1988; Whitehead, 1988; Lasiter et al., 1989). Sections were then incubated in PBS containing 0.4% Triton X-100 for 1 h and then placed in PBS containing 0.2% Triton X-100 with 1:400 streptavidin Alexa Fluor 488 (Jackson ImmunoResearch; 016-540-084, RRID: AB_2315383) for 2 h to visualize the BDA-filled NST relay cells. The CT terminal fields were labeled with TMR and did not require further processing for visualization. Sections were mounted onto gel-coated slides and coverslipped with Vectashield Hard Mounting Medium (Vector Laboratories).

To verify our stereotaxic injection parameters, one mouse from each group was injected and processed as described above. However, these brains were sectioned coronally at 100 μm to allow for viewing of the injection tract as well as the center of the injection (Fig. 1*A*).

### Confocal microscopy

Tissue was imaged using a Nikon 80i microscope fitted with a Nikon C2 scanning system (Nikon Instruments, Inc.). NST relay cells were located and imaged using a 10× objective (Nikon, CFIPlanApo; NA = 0.45) followed by a higher-resolution image taken with a 63× oil-immersion objective (Nikon, PlanApo VC; NA = 1.4). The fluorescent labels were matched to the wavelengths of the respective lasers used to image the tissue (argon laser – 488 nm, 10 mW, NST relay cells; DPSS laser – 561 nm, 10 mW, CT). Sequential optical sections were captured every 1 μm through the extent of every imaged cell. Images were obtained with settings adjusted so that pixel intensities were near (but not at) saturation.

### Data analysis

Confocal images were imported into Neurolucida 11.11.12 (MBF Bioscience; RRID:SCR_001775) and reconstructions were made by tracing the dendritic architecture and cell bodies throughout the *z*-stack. These tracings were then opened in Neurolucida Explorer 2.7 (MBF Bioscience; RRID:SCR_001775), and measures were taken on the dendritic architecture of each retrogradely-filled NST relay cell tracing. These measures included the number of primary dendrites, number of dendritic branch points, number of dendritic endings, dendritic length, mean dendritic length, and the dendritic complexity index (DCI). The DCI was determined from the following equation, DCI = (sum of branch tip order + number of dendritic endings) × (total dendritic length/number of primary dendrites) (Pillai et al., 2012).

In addition to collecting basic measures of dendritic architecture, we also performed Sholl analyses (Sholl, 1956) on each of the traced NST relay cells using Neurolucida Explorer 2.7 (MBF Bioscience; RRID:SCR_001775). A series of concentric rings were placed over the traced neuron, centered on the cell body. The centermost ring had a diameter of 20 μm (the diameter of the largest possible NST relay cell soma) while each of the concentric rings successively increased their diameter by 5 μm.

Finally, we calculated a polar histogram for each traced cell using Neurolucida Explorer 2.7 (MBF Bioscience; RRID:SCR_001775). This technique displays the length and direction of dendritic processes in a two-dimensional format. These polar histograms show the total length of dendrites present within 20° bins, covering 360° in the horizontal plane. To normalize for the angle that horizontal brain sections were mounted onto slides, a straight line was drawn from the bottom to the top of the fourth ventricle (or along the midline whenever the ventricle was not present). Representative tracings were then rotated as needed until the line was oriented vertically. We then reflected all cells from the left NST 180° horizontally (i.e., along the rostral-caudal axis of the brain). The resulting datasets were a collection of cells from the left and the right NST.

### Analysis of relay cells along the dorsal-ventral axis of the NST

**Figure 2.**
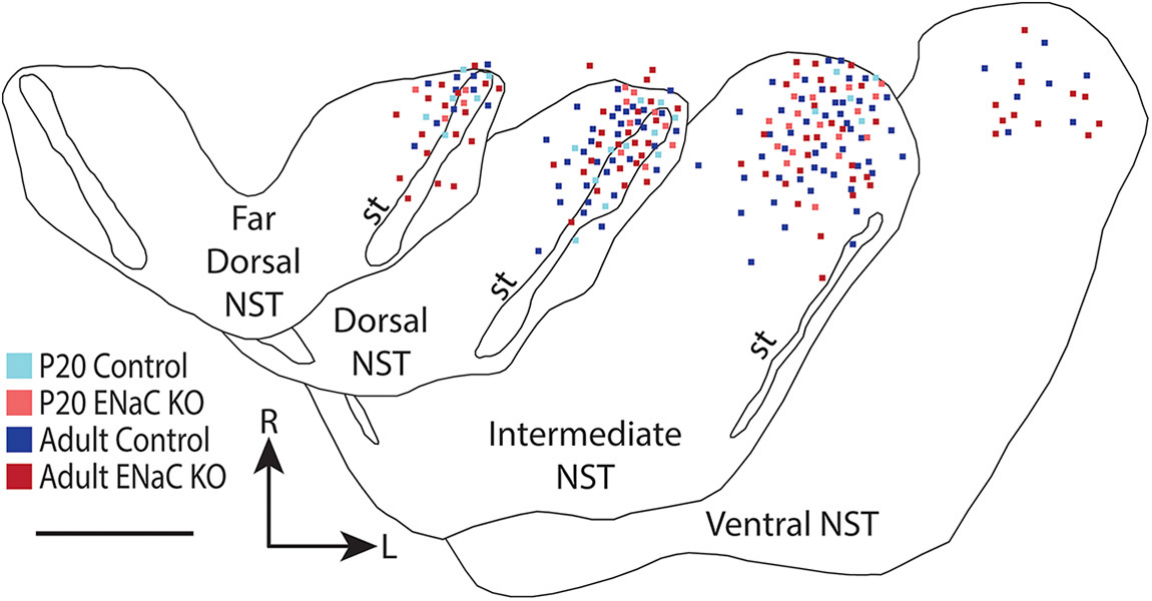
Relay cell locations throughout the dorsal ventral axis of the NST. The location of each cell from P20 control (light blue), adult control (dark blue), P20 ENaC KO (light red), and adult ENaC KO (dark red) mice are projected onto representative tracings of the horizontally-sectioned NST. The far dorsal, dorsal, intermediate, and ventral NST were located 100–300mm, 300–500mm, 500–800mm and 800–1200mm from the surface of the brainstem, respectively. Scale bar, 500mm; NST, nucleus of the solitary tract; st, solitary tract; R, rostral; L, lateral.

To assess and compare the distributions of relay cells within the NST, we recorded and plotted the location of a majority of the retrogradely-filled relay cells (231 of 257) onto representative horizontal brainstem tracings. We were not able to reliably analyze the location of 26 relay cells because of significant degradation of the tissue. To be consistent with previous literature, cells were grouped into four zones based on the dorsal-ventral axis of the NST in which they were located. The landmarks used to define these four zones were similar to what has been reported previously in mouse (Sun et al., 2015, 2017; Skyberg et al., 2017) and rat (King and Hill, 1991; Krimm and Hill, 1997; May and Hill, 2006; Sollars et al., 2006; Mangold and Hill, 2008; Corson and Hill, 2011). The first zone, the far dorsal zone of the NST, is characterized by sections with a relatively large fourth ventricle, a small solitary tract in the rostral portion of the NST, and by a lack of the hypoglossal and facial nucleus. The far dorsal zone is located between 100 and 300 μm from the surface of the brainstem in mouse (Fig. 2). The dorsal zone also contains the fourth ventricle but, in these sections, it does not extend as far in the medial-lateral plane as in the far dorsal zone. Sections from the dorsal zone have an NST that extends more rostrally than in the far dorsal zone, a solitary tract that occupies nearly the entire rostral-caudal extent of the NST, and the presence of the dorsal cochlear nucleus. The dorsal zone is immediately dorsal to the hypoglossal nucleus and is located roughly 300–500 μm from the surface of the brainstem (Fig. 2). The intermediate zone is characterized by a significant thinning of the fourth ventricle compared with the far dorsal and dorsal zones, the solitary tract is thinner and shorter than seen in the dorsal zone, the rostral pole of the NST extends further anteriorly than in the dorsal zone, and both the hypoglossal and facial nuclei are evident. The intermediate zone is located around 500 and 800 μm from the surface of the brainstem (Fig. 2). The ventral zone is at a level immediately ventral to the fourth ventricle, the NST is less defined and narrow at its rostral-most extent compared with more dorsal zones, the ventral cochlear nucleus is apparent, and the hypoglossal and facial nuclei are evident. The ventral zone contains the remainder of the NST and is located roughly 800 and 1200 μm from the surface of the brainstem (Fig. 2). It should be noted that the NST is oriented within the brainstem with the caudal-most portion of the NST positioned dorsal to the rostral-most portion of the NST (Ganchrow et al., 2014). That is, the NST extends rostrally and ventrally from the caudal-most extent of the NST. Therefore, the far dorsal zone more accurately represents the dorsal-caudal portion of the NST and the ventral zone represents the ventral-rostral portion of the NST.

### CT nerve neurophysiology

To establish that KO of the *Scnn1a* gene in the tongue resulted in reduced functional CT responses to NaCl and to determine the normal development of ENaC function in control mice, we recorded taste-stimulus-evoked whole-nerve activity from the CT of each group of mice using methods described previously (Sun et al., 2015, 2017; Skyberg et al., 2017). Briefly, mice were anesthetized as described for the retrograde parabrachial injection procedure. The animals were then tracheotomized and placed on a circulating water heating pad to maintain body temperature. Hypoglossal nerves were transected bilaterally to prevent tongue movement, and the mouse was placed in a nontraumatic head holder. The left CT nerve was isolated using a mandibular approach. The nerve was exposed near the tympanic bulla, cut, desheathed, and positioned on a platinum electrode. A second electrode was placed in nearby muscle to serve as a reference. Kwik-Sil (World Precision Instruments) was placed in the cavity around the nerve to maintain a highly functioning preparation.

#### Stimulation procedure

All chemicals were reagent grade and prepared in artificial saliva (Hellekant et al., 1985). Neural responses from the CT were recorded to ascending concentrations series of 0.05, 0.1, 0.25, and 0.5 m NaCl to assess the taste responses to prototypical stimuli that represent salty to humans. Concentration series were bracketed by applications of 0.5 m NH_4_Cl to monitor the stability of each preparation and for normalizing taste responses. Solutions were applied to the tongue in 5 ml aliquots with a syringe and allowed to remain on the tongue for ∼20 s. We used this period of stimulation so that we could measure the steady state responses. After the application of each solution, the tongue was rinsed with artificial saliva for ≥1 min (Hellekant et al., 1985). This period allowed a full recovery of neural responses (i.e., the responses were not adapted by previous responses; Shingai and Beidler, 1985). In addition, responses to the NaCl concentration series were recorded in presence of the ENaC blocker, amiloride (50 μm). Rinses during this series were with amiloride in artificial saliva.

All responses were calculated as follows: the average voltage of the spontaneous activity that occurred for the second before stimulus onset was subtracted from the voltage that occurred from the period from the first to sixth second after stimulus application. Response magnitudes were then expressed as ratios relative to the mean of 0.5 m NH_4_Cl responses before and after taste stimulation. Whole-nerve response data were retained for analysis only when 0.5 m NH_4_Cl responses that bracketed a concentration series varied by <10%.

### Experimental design and statistical analyses

All measurements presented are given as means (±SEM). Prism 7 (GraphPad; RRID:SCR_002798) was used for quantification and statistical analysis.

#### Measurements of NST relay cell location and dendritic morphology

Experiments investigating NST relay cell dendritic architecture were conducted in control and αENaC KO mice at P18–P22 (P20 control, four mice, two male/two female; P20 αENaC KO, three mice, two male/one female) and P60–P120 (adult control, eight mice, three male/five female; adult αENaC KO, 10 mice, four male/six female). Neurons collected from different mice within the same age × experimental group were collapsed into a single group, resulting in four larger neural populations (22 cells from P20 control mice, 31 cells from P20 αENaC KO mice, 123 cells from adult control mice, 81 cells from adult αENaC KO mice). The χ^2^ test was used to compare the proportion of bipolar and multipolar cells in both animal groups at each age as well as to compare the distribution of relay cells along the dorsal-ventral axis of the NST between control and αENaC KO mice at both ages. A one-way ANOVA was used to statistically compare measures of dendritic architecture (number of primary dendrites, number of dendritic branches, total dendritic length, dendritic complexity) between control and αENaC KO mice at P20 and adulthood. To statistically compare the mean frequency and mean length of each order of dendrite between control and αENaC KO mice at P20 and adulthood, a two-way ANOVA was used (four groups × seven dendritic orders). Two-way ANOVAs were also used to compare measurements collected from Sholl analyses of cells from control and αENaC KO mice at P20 and adulthood (four group × 47 distances from cell soma). We used the Bonferroni procedure to adjust the α level for multiple comparisons (Armstrong, 2014); *p* ≤ 0.05 were regarded as statistically significant.

#### Total CT terminal field volumes

Experiments inspecting the total CT terminal field volume were done in control and αENaC KO mice at P20 (P20 control, six mice, three male/three female; P20 αENaC KO, three mice, three male/0 female) and P60–P120 (adult control, seven mice, three male/four female; adult αENaC KO, six mice, four male/two female). The mean (±SEM) total CT terminal field volume was calculated for control and αENaC KO mice at P20 and adulthood, and a one-way ANOVA was used to statistically compare these means. The Bonferroni procedure was used to adjust the α level for multiple comparisons (Armstrong, 2014), and only *p* ≤ 0.05 was considered to be statistically significant.

#### CT whole-nerve neurophysiology

Experiments examining CT whole-nerve taste responses were done in control and αENaC KO mice at P20 (P20 control, three mice, two male/one female; P20 αENaC KO, two mice, one male/one female) and P60–P120 (adult control, five mice, three male/two female; adult αENaC KO, three mice, one male/two female). Due to the small number of observations in each group, we did two statistical tests that are most appropriate for these types of comparisons. For both tests, we focused on group- and age-related differences at 0.25M NaCl and 0.5M NaCl. Taste responses to lower concentrations showed no differences between control and ENaC KO mice at either age. First, we compared mean (± SEM) relative CT responses to 0.25M and 0.5M NaCl (normalized to 0.5M NH4Cl responses) between P20 control and P20 ENaC KO mice and between adult control and adult ENaC KO mice with multiple t-tests, and with the alpha level (0.05) corrected by the Holm-Sidak correction method. We also did the Mann-Whitney nonparametric test for the same comparisons. Both statistical methods provided the same outcome. We present only the statistical findings from the multiple t-test comparisons.

## Results

BDA injections into the waist region of the PBN resulted in Golgi-like, retrograde labeling of relay cells in the NST (Fig. 1). For the relay cells from the P20 control mice (*n* = 22), only two cells (9.10%) were bipolar, while 20 (90.9%) were multipolar. Similarly, of the 123 relay cells from adult control mice, only 16 (13.0%) were bipolar while 107 cells (87.0%) were multipolar. The relay cell populations in P20 αENaC KO and adult αENaC KO mice had similar proportions of bipolar and multipolar cells as the control sample. Of the relay cells from P20 αENaC KO mice (*n* = 31), three cells (9.70%) were bipolar while 28 cells (90.3%) were multipolar. Of the 81 relay cells from adult αENaC KO mice, six (7.40%) were bipolar and the remaining 75 (92.6%) were multipolar. Statistical analysis using χ^2^ analysis confirmed that the distribution of bipolar and multipolar cells in all four groups were not statistically different from each other (χ^2^(3) = 1.72, *p* = 0.634). These distributions were comparable to what has been reported in another study of rodent NST relay cell morphologies (Corson and Erisir, 2013). See Figure 1*D–G* for representative cell tracings from P20 control (Fig. 1*D*), adult control (Fig. 1*E*), P20 αENaC KO (Fig. 1*F*), and adult αENaC KO mice (Fig. 1*G*).

There was variability in the number of labeled cells that we were able to collect from each mouse, especially within the adult control group (Extended Data Fig. 3-1*A*) Despite this variability, a one-way ANOVA did not find a significant difference in the average number of cells labeled between groups Extended Data Fig. 3-1*A*, *F*_(3,24)_ = 2.849, *p* = 0.062; ). We note that this statistical test was near but not at the threshold for statistical significance.

**Figure 3.**
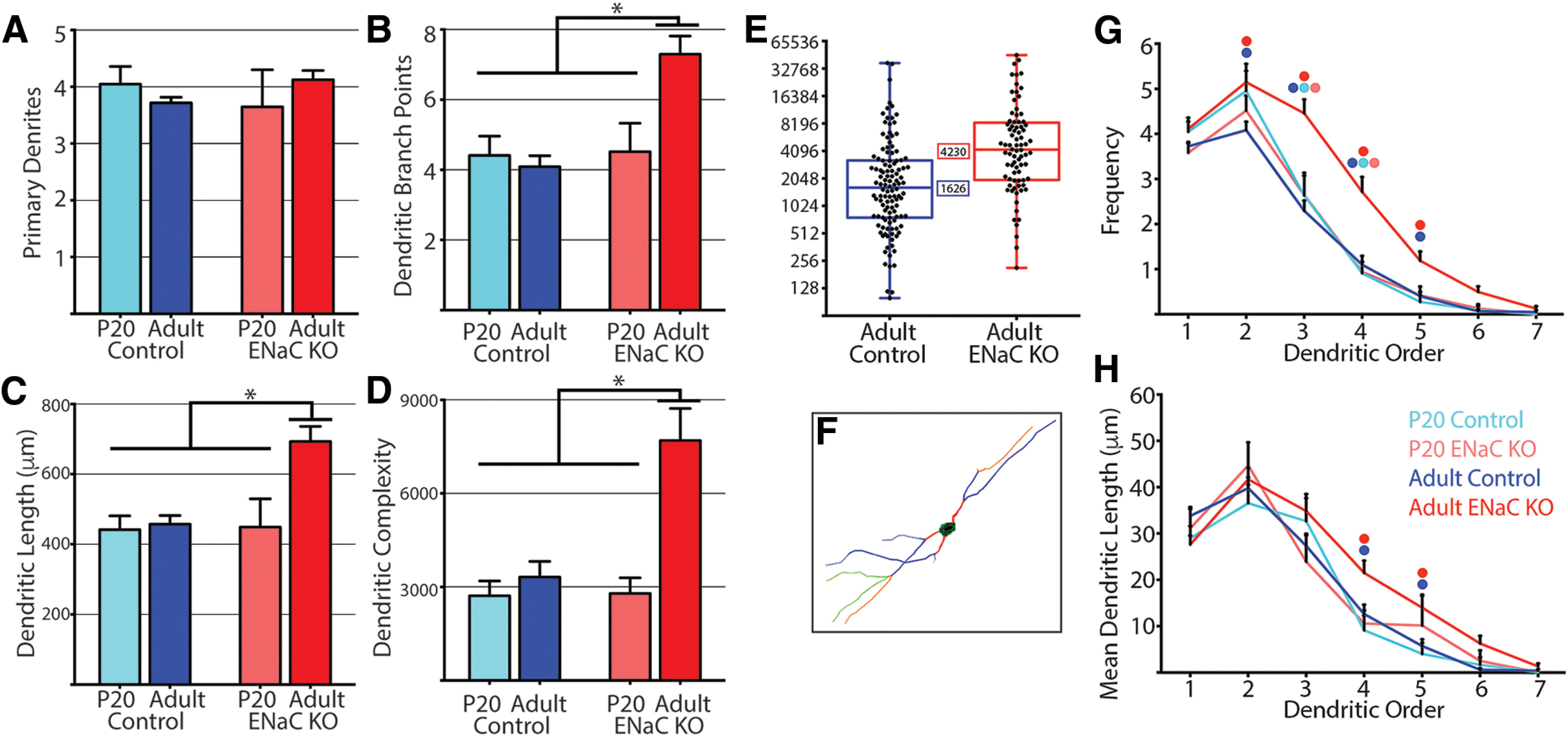
Dendritic characteristics of NST relay cells. ***A***, Mean (± SEM) number of primary dendrites in relay cells from P20 control (light blue), adult control (dark blue), P20 ENaC KO (light red), and adult ENaC KO (dark red) mice. ***B***, Mean (± SEM) number of dendritic branch points in relay cells from P20 control (light blue), adult control (dark blue), P20 ENaC KO (light red), and adult ENaC KO (dark red) mice. ***C***, Mean (± SEM) total dendritic length (mm) from relay cells from P20 control (light blue), adult control (dark blue), P20 ENaC KO (light red), and adult ENaC KO (dark red) mice. ***D***, Mean (± SEM) dendritic complexity (see methods for details) of relay cells from P20 control (light blue), adult control (dark blue), P20 ENaC KO (light red), and adult ENaC KO (dark red) mice. Lines and brackets above B, C and D denote comparisons that reached statistical significance using a one-way ANOVA while correcting for multiple comparisons * *p* < 0.05. ***E***, Box and whisker plot of dendritic complexity measure for adult control and adult ENaC KO mice, inset box denotes the median for each group of cells. ***F***, Diagram illustrating the dendritic order characterization scheme used where primary dendrites are red, second-order dendrites are blue, third-order dendrites are orange, and fourth-order are green. G-H, Dendritic characteristics by dendritic order. ***G***, Average (± SEM) number of dendrites by dendritic order in relay cells from P20 control (light blue), adult control (dark blue), P20 ENaC KO (light red), and adult ENaC KO (dark red) mice. ***H***, Mean (± SEM) dendritic lengths by dendritic order in relay cells from P20 control (light blue), adult control (dark blue), P20 ENaC KO (light red), and adult ENaC KO (dark red) mice. Colored circles above each data point denote groups that are significantly different from each other (*p* < 0.05). Also see Extended Data Figures 3-1 and 3-2.

10.1523/ENEURO.0140-20.2020.f3-1 Extended Data Figure 3-1 Average dendritic measurements for each animal. A, Number of NST relay cells labeled in each animal used in this study. Each dot refers to the number of cells labeled in one animal while the bars indicate the group means (± SEM). B, Average number of dendritic branch points occurring in neurons from one animal. Each dot reflects the average of each mouse while the bars indicate the group means (± SEM). C, Same as B but for average dendritic length. D, Same as B but for average dendritic complexity. Download Figure 3-1, TIF file.

### Distribution of relay cells within the NST along the dorsal-ventral axis

Figure 2 shows the location of 231 of the 257 retrogradely-labeled relay cells in the NST, as defined with horizontal sections (Sun et al., 2015, 2017; Skyberg et al., 2017). Relay cells were located throughout the dorsal ventral axis of the NST. Approximately 37 (16.0%) cells, regardless of group, were located in the far dorsal zone of the NST. The dorsal and intermediate zones contained a large majority of retrogradely-labeled relay cells, 77 (33.3%) cells and 98 (42.4%) cells, respectively. Finally, the ventral zone only contained 19 (8.30%) retrogradely-labeled cells. This distribution reflects the relative percentage of terminal field label from the CT in control mice (Zheng et al., 2014; Skyberg et al., 2017; Sun et al., 2017).

The dorsal-ventral distribution of relay cells from adult control mice was not significantly different from adult ENaC KO mice (χ^2^(3) = 7.44, *p* = 0.06). However, when we compared the dorsal-ventral distribution of relay cells from the P20 control population with that of the adult control distribution, we found a statistically significant difference (χ^2^(3) = 9.89, *p* = 0.02). The distribution of relay cells from P20 control mice was shifted to the far dorsal (27.0% in P20 controls vs 13.0% in adult controls) and dorsal (50.0% P20 controls vs 29.0% in adult controls) sections when compared with that in adult control mice. The distribution in P20 control mice also did not have relay cells in the ventral sections of the NST, skewing the distribution toward the dorsal sections. Thus, relay cells from the P20 and adult control populations were distributed differently along the dorsal-ventral axis of the NST, suggesting an alteration in the region-specific projection pattern from the NST to the PBN throughout development. However, this may also be because of the large differences in the number of cells in each of these control groups. Finally, the dorsal-ventral distribution of P20 ENaC KO relay cells was not significantly different from that of the adult ENaC KO group (χ^2^(3) = 3.24, *p* = 0.356). Thus, despite the large differences in the number of cells in these two neuronal populations in ENaC KO mice, they are sampled similarly along the dorsal-ventral axis of the NST (Fig. 2).

### Relay cells from adult αENaC mice have more complex dendritic fields

There were no significant differences between the mean (±SEM) number of primary dendrites of relay cells among P20 control (4.05 ± 0.3), adult control (3.72 ± 0.1), P20 αENaC KO (3.65 ± 0.7), and adult αENaC KO (4.12 ± 0.2) mice (*F*_(3,253)_ = 1.15, *p* = 0.330; Fig. 3*A*). Likewise, the mean number of primary dendrites of only the multipolar cells in each group was not significantly different among these groups (*F*_(3,226)_ = 1.78, *p* = 0.151; data not shown). Thus, the number of primary dendrites of NST relay cells did not change throughout development and was not influenced by the absence of sodium taste activity during development.

10.1523/ENEURO.0140-20.2020.f3-2 Extended Data Figure 3-2 Dendritic complexity for every cell used in this study organized by mouse from which it came. A, Dendritic complexity of all neurons from the 4 P20 control mice. B, Dendritic complexity of all neurons from the 3 P20 ENaC KO mice. C, Dendritic complexity of all neurons from the 8 adult control mice. D, Dendritic complexity of all neurons from the 10 adult ENaC KO mice. Dots indicate dendritic complexity of a single cell while the bars indicate the mean dendritic complexity of all the cells from one animal. The dendritic complexity of one adult ENaC KO cell fell outside of the bounds of the y axis. This cell is represented by a red dot and its dendritic complexity measure is to the right of the red dot. Download Figure 3-2, TIF file.

While the number of primary dendrites was not affected by the removal of ENaC-mediated sodium taste activity, we found that the number of dendritic branch points was affected. The mean (±SEM) number of branch points was 1.6–1.8× greater in cells from adult αENaC KO mice (7.30 ± 0.5) compared with cells from P20 control (4.41 ± 0.6), adult control (4.09 ± 0.3), and P20 αENaC KO mice (4.52 ± 0.8). This difference was significant (*F*_(3,253)_ = 11.5, *p* < 0.0001; Fig. 3*B*). The mean number of dendritic branch points of relay cells from P20 control, adult control, and P20 αENaC KO mice were not significantly different from each other (*p* > 0.05; Fig. 3*B*). Therefore, the variability in the mean number of dendritic branch points of all neurons among individual mice is not responsible for the significant group-related differences we report for dendritic branch points (Extended Data Fig. 3-1*B*).

Similar to the number of dendritic branch points, the total dendritic length of relay cells from adult αENaC KO mice was significantly greater than that of any other group (*F*_(3,253)_ = 9.59, *p* < 0.0001; Fig. 3*C*). The mean (±SEM) dendritic length (μm) in cells from adult αENaC KO (692.76 μm ± 43.0) mice was 1.5–1.6× larger than that of cells from P20 control (441.51 μm ± 39.4), adult control (457.29 μm ± 24.7) and P20 αENaC KO mice (448.95 μm ± 80.6). The mean dendritic length in relay cells in P20 control, adult control, and P20 αENaC KO mice were not different from each other (*p* > 0.05; Fig. 3*C*). Calculating the mean dendritic length of all neurons from any one mouse illustrates that, while there is some variability from animal to animal, the significant differences we report are not due solely to one or two animals (Extended Data Fig. 3-1*C*).

These large differences in the mean dendritic length and number of dendritic branch points in the adult αENaC KO relay cell population resulted in a similar difference in the mean DCI (Fig. 3*D*). The DCI is a comprehensive measurement of the complexity a cell’s dendritic field (see methods for details). The mean (±SEM) DCI in relay cells in adult αENaC KO mice (7692.13 ± 1032.6) was 2.3–2.8× larger than that of relay cells in P20 control (2716.15 ± 475.0), adult control (3314.65 ± 502.6), and P20 αENaC KO (2790.28 ± 501.2) mice (Fig. 3*D*). These differences were statistically significant (*F*_(3,253)_ = 8.84, *p* < 0.0001). There were no significant differences among the mean DCI of relay cells from P20 control, adult control, and P20 αENaC KO mice (*p* > 0.05; Fig. 3*D*). Figure 3*E* shows a box and whisker plot comparison of the DCI for both adult populations of relay cells (P20 populations were not included because of small number of cells labeled in each population). A large majority of relay cells from adult ENaC KO mice (81.5%) had a DCI larger than the median DCI of the adult control population. Calculating the mean dendritic complexity of all neurons from any one mouse shows that while there is variability from animal to animal, the significant differences we report are not driven by a few outliers (Extended Data Fig. 3-1D, Extended Data Fig. 3-2).

### Relay cells from adult αENaC mice have higher-order dendrites

The results above suggest that the increased complexity observed in relay cells from adult αENaC KO mice are because of changes in the number and/or length of higher-order dendrites (for illustration of dendritic order, see Fig. 3*F*). To study this, we calculated the mean frequency (Fig. 3*G*) and mean dendritic length (Fig. 3*H*) of each order of dendrite for each group of cells. Statistical analysis found a significant effect of animal group on mean frequency (*F*_(3,1771)_ = 35.7, *p* < 0.0001; Table 1) and mean dendritic length (*F*_(3,1771)_ = 5.12, *p* = 0.002; Table 1).

**Table 1:** Significant results

Order of dendrite	Significant comparisons	Number of dendrites *p* values	Mean dendritic length *p* values
		*F*_(6,1771)_ = 193.5	*F*_(6,1771)_ = 98.81
First order	None	No significance	No significance
Second order	Adult αENaC KO, adult control	*p* < 0.0001	*p* = 0.9816
	Adult αENaC KO, adult control	*p* < 0.0001	*p* = 0.0514
Third order	Adult αENaC KO, P20 control	*p* < 0.0001	*p* = 0.9982
	Adult αENaC KO, P20 αENaC KO	*p* < 0.0001	*p* = 0.0569
	Adult αENaC KO, adult control	*p* < 0.0001	*p* = 0.0117
Fourth order	Adult αENaC KO, P20 control	*p* < 0.0001	*p* = 0.0593
	Adult αENaC KO, P20 αENaC KO	*p* < 0.0001	*p* = 0.0575
Fifth order	Adult αENaC KO, adult control	*p* = 0.0085	*p* = 0.0225
Sixth order	None	No significance	No significance
Seventh order	None	No significance	No significance

Consistent with what was reported above, there were no differences in the mean number of primary dendrites among neurons from P20 control, adult control, P20 αENaC KO, and adult αENaC KO mice (Fig. 3*E*; Table 1). There were also no differences in the mean dendritic lengths of primary dendrites among neurons in P20 control, adult control, P20 αENaC KO, or adult αENaC KO mice (Fig. 3*F*; Table 1).

As was predicted, the Bonferroni *post hoc* test found that relay cells from adult αENaC KO mice had significantly more second-order (1.26×), third-order (1.94×), fourth-order (2.47×), and fifth-order (2.98×) dendrites than cells from adult control mice (Fig. 3*G*; Table 1). However, the mean dendritic lengths of these dendritic groups were only significantly different when comparing fourth and fifth-order dendrites (Fig. 3*H*; Table 1). Fourth-order and fifth-order dendrites were 1.70× and 2.46× longer in cells from adult αENaC KO mice than in cells from adult control mice. The mean dendritic length of third-order dendrites between adult αENaC KO and control mice was just above the threshold for significance (Table 1).

Adult αENaC KO mice also had more third-order and fourth-order dendrites than both the P20 control (1.79× and 2.99×, respectively) or P20 αENaC KO (1.69× and 2.90×, respectively) populations (Fig. 3*G*; Table 1). The mean dendritic lengths of these dendrites were not significantly different between any of these groups (Fig. 3*H*; Table 1).

There were no differences in the frequency or mean dendritic lengths associated with sixth-order or seventh-order dendrites among any groups of relay cells (Fig. 3*G*,*H*; Table 1). This was due, in part, to very few cells having sixth-order or seventh-order dendrites, regardless of group.

### Gross dendritic morphology and orientation of relay cells from adult αENaC KO mice appear normal despite increased dendritic complexity

**Figure 4.**
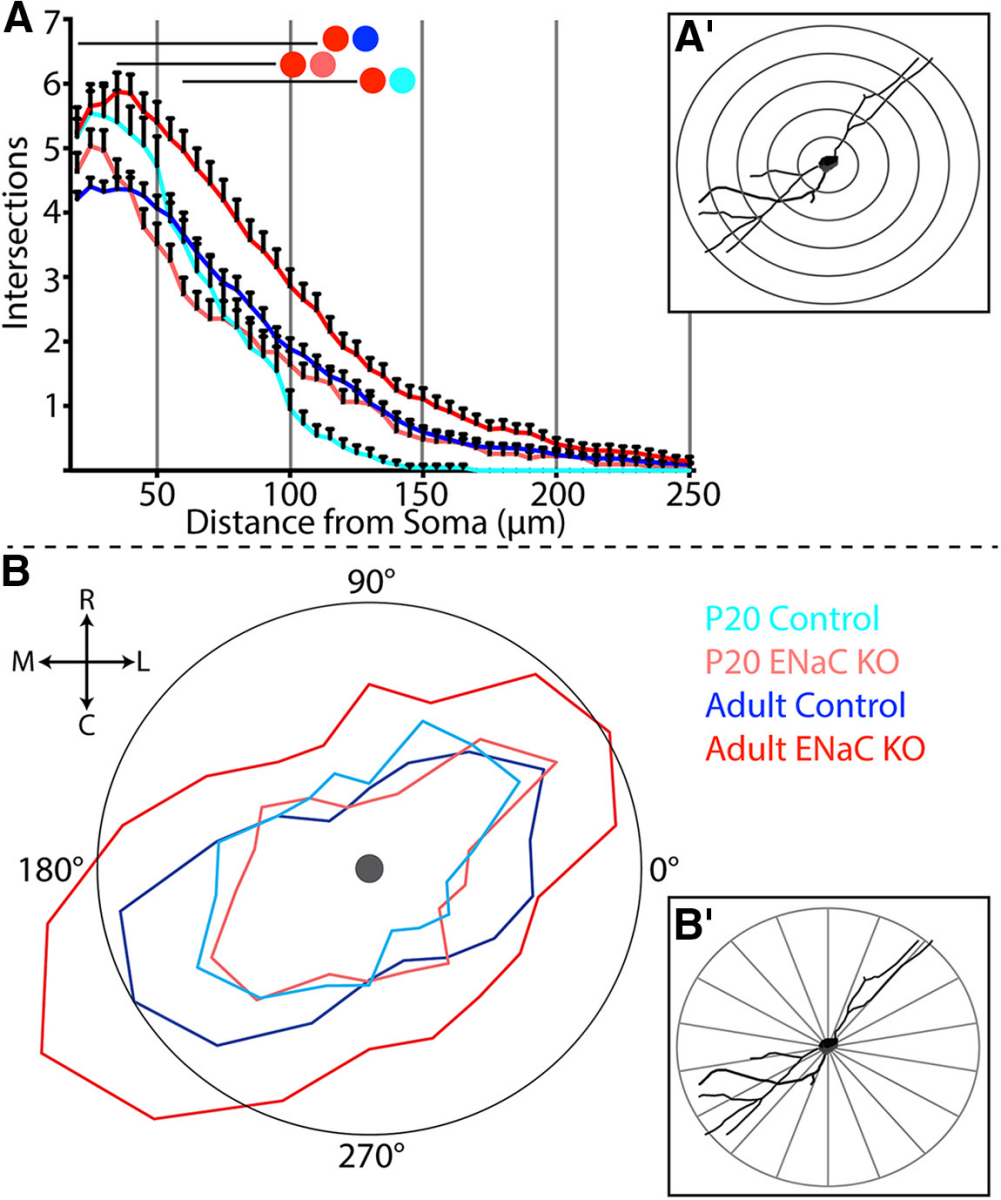
Spatial organization of dendritic fields. ***A***, Sholl analysis measuring the number of intersections created by dendrites of NST relay cells from P20 (light blue), adult control (dark blue), P20 ENaC KO (light red), and adult ENaC KO (dark red) mice as dendrites extend away from the cell soma. Colored circles with lines denote areas of significant difference (*p* < 0.05) between two groups. ***A′***, Representation of how the dendritic field is divided with a sholl analysis. ***B***, P Polar histogram showing the angular distribution of dendritic material in the horizontal plane of cells from P20 control (light blue), adult control (dark blue), P20 ENaC KO (light red), and adult ENaC KO (dark red) mice. Area around the cell soma (grey circle) was divided into 20° bins as depicted in ***B′***. The total amount of dendritic material within each 20° bin was measured for each cell and then averaged across each cell group. Mean dendritic lengths for each group within each 20° bin are represented on a relative scale by the distance each point is from the center of the soma. The adult control bin with the largest mean dendritic length (200–220°) was used to normalize all of the other data points. Thus, the black circle represents a normalization ratio of 1. R, rostral; C, caudal; L, lateral; M, medial.

To assess how the increased dendritic complexity observed in relay cells from adult αENaC KO mice affected the overall dendritic morphology, we computed a Sholl analysis (for details, see Materials and Methods; Fig. 4*A’*). Briefly, Sholl analyses use a series of concentric circles, centered on the cell soma, to assess how dendritic architecture changes as the dendrites extend from the cell soma (Fig. 4*A’*). The number of times dendrites intersect with each concentric circle (Fig. 4*A*) are then collected to assess how the dendritic field is spatially organized.

Figure 4*A* shows the results from a Sholl analysis, calculating the mean number of times dendritic material intersected with each concentric ring for each group of NST relay cells. As would be predicted from the dendritic complexity results shown above, relay cells in adult αENaC KO mice averaged significantly more dendritic intersections than any other group of relay cells (*F*_(3,11887)_ = 132, *p* < 0.0001; Fig. 4*A*). However, these differences were only significant within the first 125 μm from the cell soma. Group-related differences were evident at adulthood. The number of dendritic intersections for cells in adult αENAC KO mice were significantly greater than that from adult control mice, with differences occurring 20–110 μm from the cell soma (*p* < 0.05; Fig. 4*A*). Moreover, we found that the number of dendritic intersections increased after P20 in αENaC KO mice, with age-related differences occurring 35–95 μm from the soma (*p* < 0.05; Fig. 4*A*). Finally, the dendritic intersections of relay cells from the soma of adult αENaC KO mice were significantly greater than from P20 control mice (60–125 μm; *p* < 0.05; Fig. 4*A*). Thus, a majority of the changes in the dendritic architecture seen in adult αENaC KO mice relay cells occurred within the first 125 μm from the cell soma.

Interestingly, while relay cells from adult αENaC KO mice had significantly more intersections than that in the adult control mice, the overall shape of the curves created in the Sholl analysis were similar, suggesting that the overall organization of dendrites of these two cell groups were similar, despite the differences in dendritic complexity and dendritic length. Likewise, comparing the shape of both curves from the two P20 groups with that of the adult control group implies that there may be a subtle developmental reorganization of dendritic material. That is, there appears to be a slight loss of dendritic material in the dendritic field closest to the cell soma coupled with a corresponding elaboration of dendritic fields further from the cell soma between P20 and adulthood.

We also asked whether the removing ENaC-mediated taste activity altered the dendritic orientation preference that these NST relay cells maintain. Typically, these relay cells orient their dendritic fields parallel to the solitary tract, the source of gustatory input from the periphery (Davis, 1988; Corson and Erisir, 2013). This results in a dendritic orientation preference that is roughly 30–45° from the horizontal (medial-lateral) axis. To assess whether ENaC-mediated neural activity impacted this orientation preference, we created polar histograms for each cell group (for details, see Materials and Methods; Fig. 4*B’*). Figure 4*B* shows the resulting normalized polar histograms from each cell group. We found that polar histograms for P20 control and P20 αENaC KO mice were similar to each other and relatively similar to that of the adult control relay cell group (Fig. 4*B*). Furthermore, both P20 groups and the adult control group had similar dendritic orientation preferences, which was roughly 45° off the horizontal (medial-lateral). Interestingly, while the adult αENaC KO relay cell polar histogram was larger than all other groups, likely reflecting their increased dendritic lengths, the overall orientation preference of these cells was similar to that of the other three groups (Fig. 4*B*). Thus, the typical orientation preference that NST relay cells display develops relatively early in an animal’s life, before P20, and does not require ENaC-mediated taste activity to occur.

### CT terminal fields develop abnormally and do not “prune” in adult αENaC KO mice

We previously reported that ENaC-mediated taste activity is necessary for both the development and maintenance of gustatory terminal fields in the NST (Skyberg et al., 2017; Sun et al., 2017). Gustatory terminal field volumes in adult αENaC KO mice, lacking ENaC-mediated taste activity throughout life, were ∼140% larger than that of adult control mice. However, terminal fields were only analyzed in adult control and ENaC-KO mice. Therefore, it is unclear whether the terminal field changes occur before or after P20, when ENaC-mediated taste experience matures. Therefore, to gain an understanding of the developmental trajectory that these terminal fields take in both control and αENaC KO mice, we labeled the CT nerve in P20 and adult control and αENaC KO mice.

**Figure 5.**
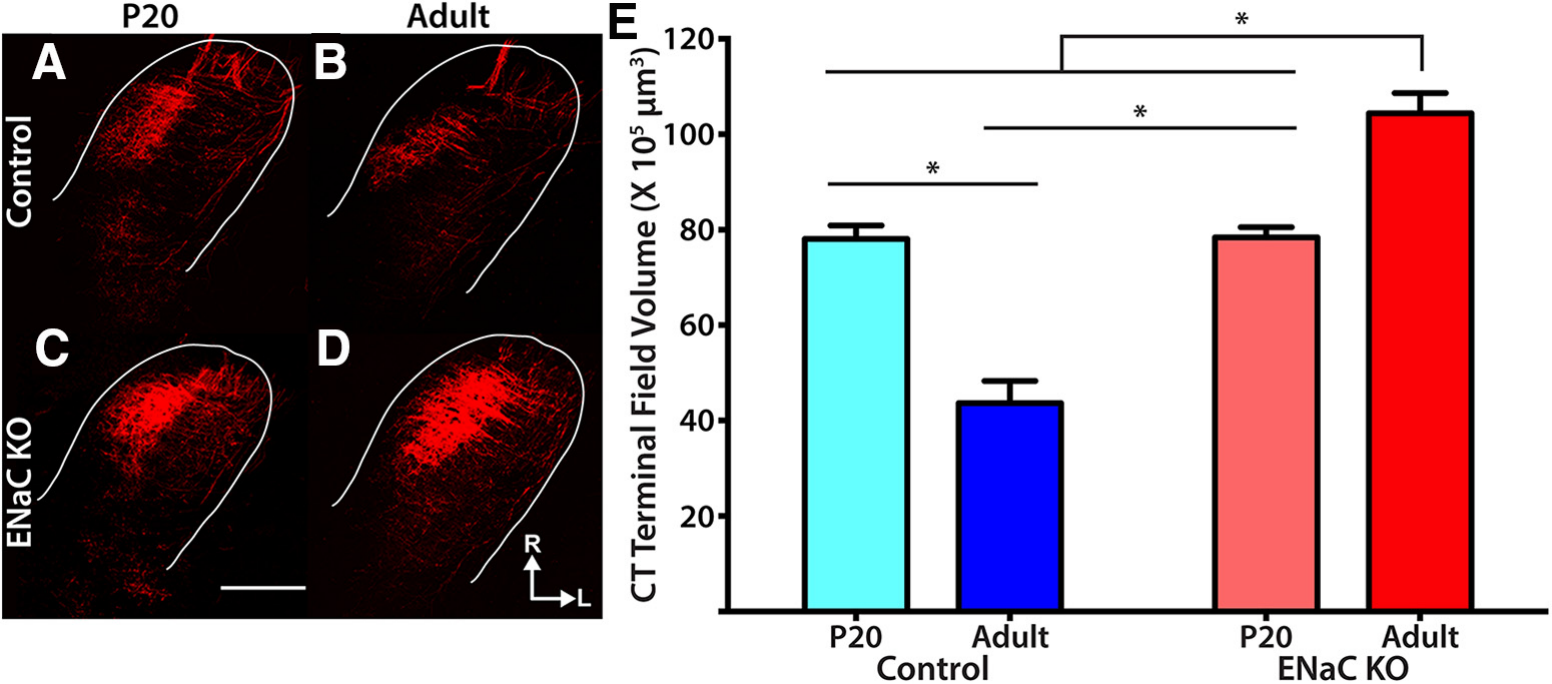
Activity-dependent development of chorda tympani (CT) terminal field. ***A-D***, Photomicrographs of the CT terminal field in horizontal sections from a P20 control (***A***), adult control (***B***), P20 ENaC KO (***C***), and adult ENaC KO (***D***) mice. White lines in ***A-D*** demark the border of the NST. ***E***, Quantification of the total CT terminal field volume in P20 control (light blue), adult control (dark blue), P20 ENaC KO (light red), and adult ENaC KO (dark red) mice. *p < 0.05. Scale bar in C, 250mm. R, rostral; L, lateral.

Figure 5 shows representative photomicrographs (Fig. 5*A–D*) of the CT as well as quantification of the total CT terminal field volume (Fig. 5*E*) for control and αENaC KO mice at the two developmental ages. Statistical analysis confirmed there was a significant difference in the total CT terminal field volume among groups (*F*_(3,18)_ = 42.7, *p* < 0.0001). Developmentally, mean CT terminal field volumes in control mice decreased with age (Fig. 5*A*,*B*,*E*). The CT terminal field volume decreased by 56% throughout development. This difference was statistically significant (*p* < 0.0001; Fig. 5*E*). This developmental decrease in CT terminal field size was similar that reported in rat (May and Hill, 2006; Mangold and Hill, 2008) and mouse (Zheng et al., 2014). By contrast, the CT terminal field volumes increased between P20 and adulthood in αENaC KO mice. That is, the mean CT terminal field volumes underwent a 33% increase with age and was statistically significant (*p* = 0.0084; Fig. 5*C–E*). These age-related differences in terminal field development resulted in dramatic differences between controls and ENaC-KO mice at adulthood. Terminal field volumes in adult αENaC KO mice were 140% larger than that found in age-matched control mice (*p* < 0.0001; Fig. 5*E*). Interestingly, the mean CT terminal field volumes in P20 control and P20 αENaC KO mice were nearly identical (*p* > 0.9999), suggesting these terminal fields develop similarly before P20, indicating that the absence of ENaC-mediated taste activity after P20 does not lead to a failure to develop (i.e., they stay at the immature state) but rather leads to increased growth of the fields.

### Adult αENaC KO mice have a loss of ENaC-mediated sodium taste activity throughout development

**Figure 6.**
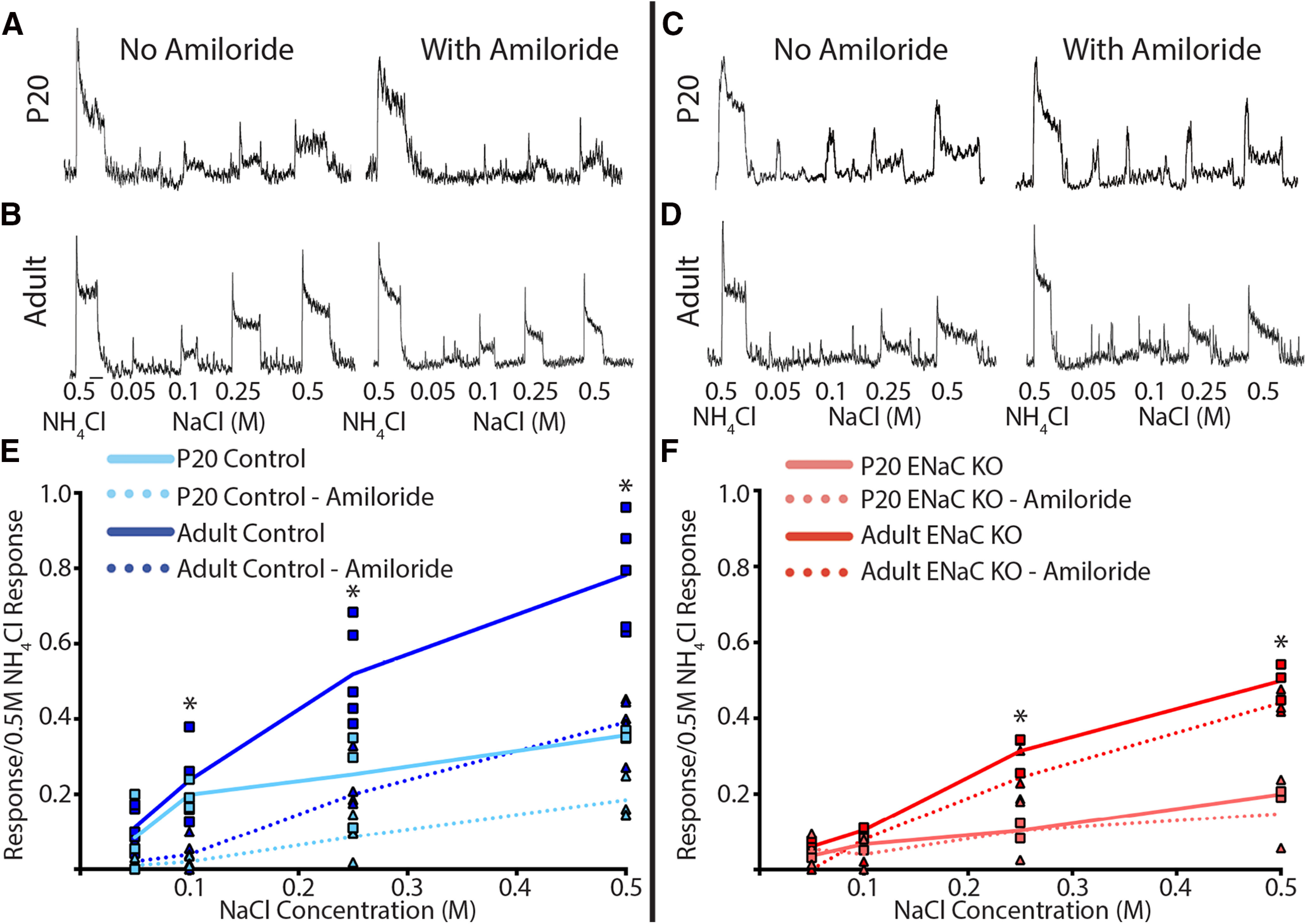
Development of chorda tympani (CT) whole nerve responses in control (left panel) and ENaC KO mice (right panel). ***A***, Integrated CT taste responses from a P20 control mouse to 0.5M NH4Cl and an increasing concentration series of NaCl (0.05, 0.1, 0.25, 0.5M) before and after lingual application of ENaC blocker, amiloride. ***B***, Same as A but from an adult control mouse. C, Integrated CT taste responses from a P20 ENaC KO mouse to 0.5M NH4Cl and an increasing concentration series of NaCl (0.05, 0.1, 0.25, 0.5M) before and after lingual application of ENaC blocker, amiloride. ***D***, Same as C but from an adult ENaC KO mouse. Scale bar in ***B***, 20s and applies to ***A-D***. ***E***, Mean Relative taste responses from P20 control mice (light blue) and adult control mice (dark blue) to a concentration series of NaCl before (solid lines) and after (dotted lines) lingual application of amiloride. Responses from individual animals before amiloride are represented by squares while responses after amiloride are represented with triangles. ***F***, Mean relative taste responses from P20 ENaC KO mice (light red) and adult ENaC KO mice (dark red) to a concentration series of NaCl before (solid lines) and after (dotted lines) lingual application of amiloride. Responses from individual animals before amiloride are represented by squares while responses after amiloride are represented with triangles. *p < 0.05.

Figure 6 shows the integrated (Fig. 6*A-D*) and normalized (Fig. 6*E*,*F*) CT whole-nerve taste responses to an increasing concentration series of NaCl in P20 control (Fig. 6*A*,*E*), adult control (Fig. 6*B*,*E*), P20 ENaC KO (Fig. 6*C*,*F*), and adult ENaC KO (Fig. 6*D*,*F*) mice. We found a clear developmental increase in the ability of NaCl to drive CT responses in control mice (Fig. 6*A*,*B*,*E*). This is particularly evident when comparing CT responses to high concentrations of NaCl. The relative responses to 0.25 and 0.5 m NaCl in P20 control mice were significantly less (40–50%) than the respective responses in adult control mice (Fig. 6*E*; t_(6)_ = 2.9–4.9; significant posttest, *p* = 0.03 and 0.005 for 0.25M NaCl and 0.5M NaCl, respectivel). Interestingly, there were also differences in NaCl responses after lingual application of amiloride between P20 control and adult control mice. Relative CT responses to 0.5 m NaCl after amiloride from P20 control mice were 50% less than the respective responses in adult control mice after amiloride (t_(6) _= 4.2; *p* = 0.01; Fig. 6*E*). Finally, while there is a developmental increase in both the amiloride-sensitive and amiloride-insensitive salt transduction pathways in control mice, the observation that amiloride attenuates NaCl responses more in adult controls than P20 controls suggests that a majority of this development is ENaC mediated (Fig. 6*E*). These findings are similar to that reported in rat (Hill and Almli, 1980; Ferrell et al., 1981; Yamada, 1980; Hill and Bour, 1985).

By comparison, conditionally deleting the *Scnn1a* gene from taste bud cells had selective effects on CT whole-nerve taste responses. Like adult control mice, increasing the concentration of NaCl in adult αENaC KO mice resulted in increased CT responses; however, the relative magnitude of these responses was lower than that of adult control mice at all stimulus concentrations (Fig. 6*E*,*F*). However, the only group-related difference occurred for taste responses to 0.5M NaCl (t_(6)_ = 15.0; *p* = 0.001 and is similar to that previously reported (Chandrashekar et al., 2010; Skyberg et al., 2017; Sun et al., 2017). Finally, while the average CT responses from P20 control mice are larger than that of P20 ENaC KO mice to any concentration of NaCl, these differences were not statistically significant (*p* > 0.05; Fig. 6*E*,*F*). This may be due in part to higher variability of P20 control mice responses, potentially reflecting a period of significant sodium taste development.

Importantly, the ENaC blocker, amiloride, did not attenuate CT responses from either P20 ENaC KO or adult ENaC KO mice (Fig. 6*C*,*D*,*F*), indicating two things. First, that removal of the *Scnn1a* gene was effective in eliminating ENaC-mediated taste activity. And second, the remaining responses in these ENaC KO mice were mediated by an amiloride-insensitive transduction pathway(s). Interestingly, both control and ENaC KO mice exhibit developmental increases in amiloride-insensitive CT whole-nerve responses between P20 and adulthood (Fig. 6), suggesting there is a postnatal development of the amiloride-insensitive salt transduction pathway in both mouse lines. Multiple studies have shown these amiloride-insensitive salt responses are dependent on the anion component of salts (chloride here; Elliot and Simon, 1990; Formaker and Hill, 1988; Ye et al., 1993; Lewandowski et al., 2016; Roebber et al., 2019). However, the transduction pathway(s) responsible for these anion responses are not as well understood. Lewandowski et al. (2016) suggest type 3 taste bud cells gate this amiloride-insensitive pathway, while Roebber et al. (2019) find a subset of type 2 taste bud cells to be responsible. Similarly, the receptor(s) mediating these amiloride-insensitive responses area also not well characterized. Regardless of the exact mechanisms, the observation that amiloride-insensitive responses develop postnatally is novel and raises interesting questions regarding the molecular/cellular changes underlying this development.

In summary, between P20 and adulthood, there is an ENaC-mediated developmental increase in the ability of NaCl to drive whole-nerve responses in control mice (Fig. 6*E*). Removal of the *Scnn1a* gene in our ENaC KO mice prevented this developmental increase after P20 in the amiloride-sensitive CT responses and prevented sodium-elicited taste activity from entering the central gustatory system (Fig. 6*F*). Finally, we found that there may also be a similar developmental change in the amiloride-insensitive pathway in both control and ENaC KO mice (Fig. 6*E*,*F*).

## Discussion

Removal of ENaC-mediated taste activity throughout life had significant effects on the development and maintenance of gustatory circuits in the mouse NST. Here, we show that the enlarged dendritic fields of gustatory relay neurons in the NST of adult αENaC KO mice parallel that found for the terminal fields of gustatory nerves that make their inputs onto these neurons. Moreover, the substantial age-related changes in terminal and dendritic field size in ENaC KO mice occur after P20, an age when ENaC-mediated peripheral taste responses begin to mature in normally-developing mice. These data suggest that there is a period of considerable reorganization in central gustatory circuity after P20 in both control mice with normal taste experience and ENaC KO mice lacking sodium taste experience. However, the direction of these changes was opposite: terminal field sizes in control mice decreased throughout early postnatal development while terminal fields and dendritic fields in ENaC KO mice increased in size. Collectively, these data are consistent with the hypothesis that peripheral taste-elicited activity short-circuits the default program driving expansion of gustatory nerve terminal fields and the dendrites of first-order central neurons that they innervate.

### Development of the first central gustatory synaptic relay in control mice

In control mice, terminal fields of gustatory afferents undergo extensive changes in size after P20. Between P15 and adulthood the CT terminal field reorganizes, resulting in a twofold decrease in terminal field volume (Fig. 5; Zheng et al., 2014). Around P30 these terminal fields reach a mature organization and are maintained throughout the animal’s life (Zheng et al., 2014). This developmental period coincides with an increase in taste response magnitudes (particularly to NaCl) in these same nerves. Interestingly, the postsynaptic dendritic fields appear fully mature well before the presynaptic terminal fields have begun their apparent activity-dependent reorganization. While a causal relationship between the development of presynaptic and postsynaptic elements of this circuit is not readily apparent, understanding changes at the synaptic level may provide useful. Wang et al. (2012) found an age-dependent decrease in the number of CT terminal synapses in the rat NST, as would be predicted by the developmental decreases in total CT terminal field volume in rat (May and Hill, 2006; Mangold and Hill, 2008) and mouse (Fig. 5; Zheng et al., 2014). Interestingly, they report a substantial developmental decrease in number of CT synapses onto GABAergic targets (Wang et al., 2012). There is also evidence that the decrease in terminal field size and numbers of synapses are paradoxically accompanied by an age-related increase in functional taste responses from the NST, especially to NaCl (Hill et al., 1983). Therefore, terminal field pruning throughout development may, in part, reflect synaptic reorganization away from GABAergic targets and toward NST relay cells. Future research measuring changes in the number, location, and strength of gustatory synapses onto NST relay cells will provide useful in further understanding the postnatal development of this circuit.

### Development of the first central gustatory synaptic relay in αENaC KO mice

Removal of ENaC-mediated sodium taste activity, beginning embryonically and continuing throughout development had multiple effects on the postnatal organization of gustatory circuits in the NST. CT terminal fields in ENaC KO mice not only failed to prune but, instead, expanded in volume into adulthood. It is not clear whether the lack of change in peripheral input around P20 in our ENaC KO mice may be driving this abnormal growth in terminal and dendritic fields or simply coincides with this dendritic expansion. Regardless of the specific mechanism, it is possible that the wholesale anatomic changes could produce profound changes in taste-related function and behavior. For example, Chandrashekar et al. (2010) report that taste-related behaviors are largely the same between ENaC KO and control mice, with the exception of responses to appetitive concentrations of NaCl. Specifically, it appears as though appetitive aspects of NaCl in salt appetite are blunted or eliminated in these mice, the normal avoidance of high concentrations of NaCl are left intact. Moreover, our findings here suggest a significant alteration in the way that brainstem taste neurons will process (i.e., code) taste stimuli.

### Role of ENaC-mediated activity in development and maintenance of the first central gustatory synaptic relay

**Figure 7.**
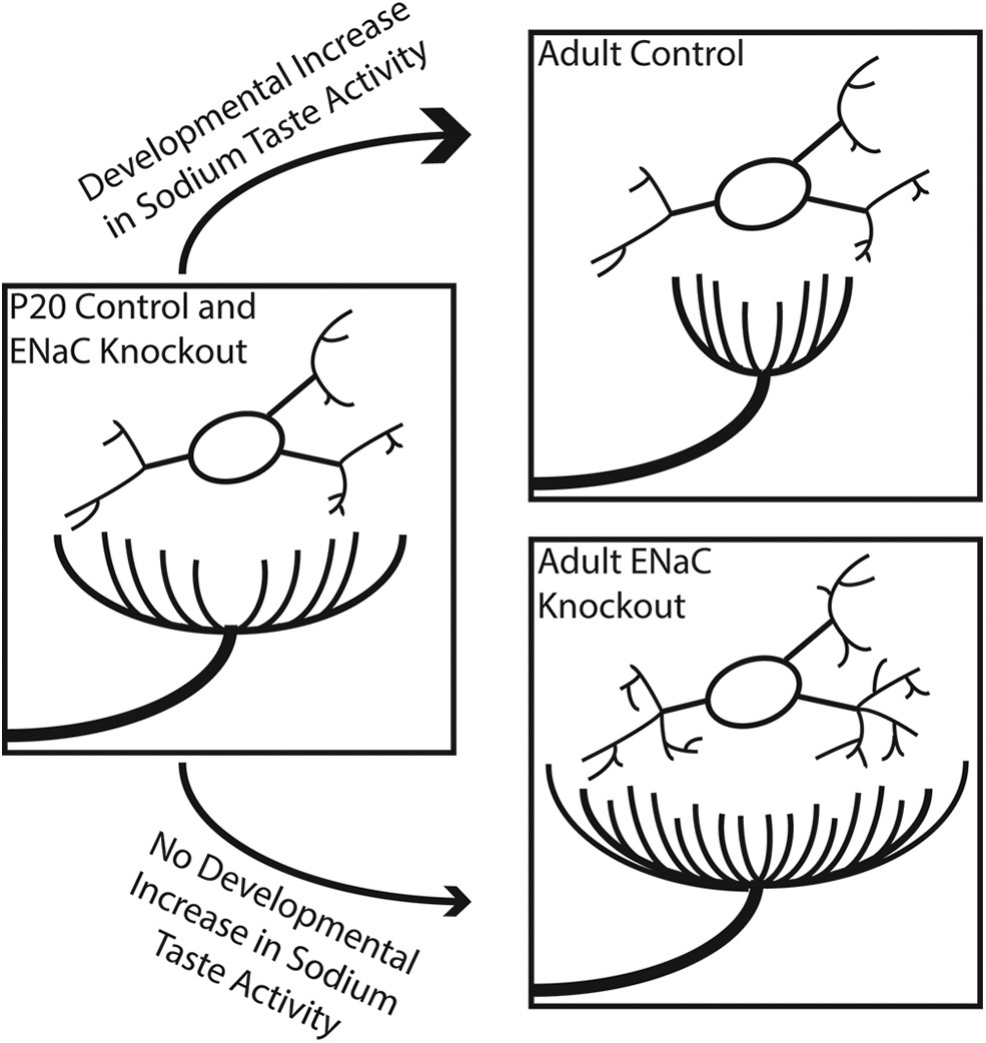
Summary figure illustrating the development of gustatory NST circuitry in control and ENaC KO mice between P20 and adulthood. In control mice, the developmental increase in sodium-taste activity drives pruning of terminal fields but does not alter NST relay cell architecture. Conversely, in ENaC KO mice that lack sodium-taste responses throughout life, gustatory terminal fields not only fail to prune but get larger. NST relay cell dendrites also became more elaborate in ENaC KO mice. Importantly, there were no differences between P20 control and P20 ENaC KO mice in terminal field size or measures of dendritic architecture.

The results from these and other experiments illustrate multiple effects of removing ENaC-mediated sodium taste activity throughout an animal’s life. Indeed, the effects of removing ENaC activity on the terminal and dendritic fields were not identical. In the case of the terminal fields of gustatory afferents, the normal developmental decrease in terminal field size was prevented in ENaC KO mice, suggesting that sodium salt activity can drive this developmental pruning of terminal fields in the NST (Fig. 7; Sun et al., 2017). We have also shown that the maintenance of mature terminal fields is dependent on ENaC-mediated sodium taste activity, without which terminal fields revert back to an immature, enlarged organization (Skyberg et al., 2017). By contrast, the dendritic fields show a different dependence on this ENaC-elicited taste activity. Because the dendritic fields resemble a mature organization by P20 in control mice, which is before ENaC-mediated activity has fully matured, it is unlikely that this reorganization is driven by ENaC activity. However, similar to terminal fields, the maintenance of NST dendritic fields after P20 appears dependent on ENaC-mediated sodium taste activity (Figs. 3, 7). Therefore, while there are differences in the specific ways ENaC activity modulates the presynaptic and postsynaptic elements of the gustatory NST circuit, removing this activity always results in an elaboration of the elements within this circuit (Fig. 7; Skyberg et al., 2017; Sun et al., 2017). One intriguing explanation for this observation is that dendritic and axonal elongation are the default growth patterns for these gustatory cells and neuronal activity attenuates this innate program. In fact, this has been proposed to occur during the morphologic development of retinogeniculate axons in the dLGN (Sretavan et al., 1988) as well as during dendritic development of Purkinje cells in the murine cerebellum (Schilling et al., 1991).

### The specificity of altered NST cells

One unique benefit of using αENaC KO mice as a model to assess the roles neural activity plays in the development and organization of gustatory circuitry is that these mice only lack taste activity from a single transduction pathway (salty; Chandrashekar et al., 2010; Skyberg et al., 2017; Sun et al., 2017). Given the specificity of our functional KO in the periphery, one may expect the central effects to exhibit some specificity as well. Put more simply, if we are only removing sodium salt taste, are we also only altering one subgroup of NST relay cells? While it is impossible to determine this conclusively without also knowing the taste response profiles of each of these cells, analysis of the dendritic complexity across adult control and ENaC KO populations is consistent with the idea that dendritic growth occurs in a majority of cells rather than one subpopulation. That is, 81.5% of ENaC KO cells (66 out of 81) had dendritic complexity measures larger than the median dendritic complexity of the adult control sample (Fig. 3*E*). Furthermore, plotting box and whisker plots for the dendritic complexity of each population shows that both the lower and upper quartile, as well as the range, are substantially higher in the adult ENaC population when compared with the adult control population (Fig. 3*E*). This is consistent with a wholesale increase in dendritic complexity rather than an increase in only one subpopulation of cells. Finally, it has been reported that the majority of rodent NST relay cells are generally broadly tuned and respond to more than one taste stimuli, indicating there may not be a large number of physiologically defined subpopulations of NST relay cells that would be specifically affected by deletion of peripheral sodium salt taste (Hill et al., 1983; Sato and Beidler, 1997; Cho et al., 2002; Carleton et al., 2010). However, a recent study has shown a population of NST relay cells that selectively respond to sour stimuli (Zhang et al., 2019), suggesting there may be subpopulation of neurons in our data that are unaffected by the functional removal of ENaC.

## Conclusion

We show here that removal sodium salt taste beginning embryonically and continuing throughout development had large effects on the development and organization of gustatory circuits in the NST. Both gustatory afferent terminal fields and the dendritic fields of NST relay cells were significantly larger in adult ENaC KO mice than in age-matched controls. Furthermore, the effects of removing sodium taste responses did not occur until after P20, which is when functional taste responses in control mice increase in magnitude. Collectively, these data suggest the earliest central synaptic relay in the gustatory system is plastic late into development and the normal formation of these circuits is directed by sufficient amounts of neural inputs that relate to sodium salt taste.

## Synthesis

Reviewing Editor: Deanna Smith, University of South Carolina Decisions are customarily a result of the Reviewing Editor and the peer reviewers coming together and discussing their recommendations until a consensus is reached. When revisions are invited, a fact-based synthesis statement explaining their decision and outlining what is needed to prepare a revision will be listed below. The following reviewer(s) agreed to reveal their identity: Alan Spector, John Boughter. Note: If this manuscript was transferred from JNeurosci and a decision was made to accept the manuscript without peer review, a brief statement to this effect will instead be what is listed below.

Both reviewers agreed this is a well written manuscript, with high quality science worthy of publication. The findings are interesting and novel, demonstrating how postnatal dendritic organization of taste projection neurons in the NTS depends on the presence of the a subunit of the aENaC in taste bud cells. The work presents an important series of findings because it provides new details of activity-dependent development of gustatory circuits in the rodent brain, opening up interesting speculation as to how far upstream these effects persist. However, both reviewers had concerns about statistical analyses, recommending that more is needed to support the use of ANOVAs, and recommending that the authors add information and analyses to the manuscript to allay any concerns regarding collapsing of neurons into a single group. There was concern that more comparison of consistency across subjects within an experimental group is needed and that parametric comparisons might not be appropriate for the electrophysiology experiments which have low subject numbers.

Please carefully consider the specific comments from each reviewer before resubmitting the manuscript:

Reviewer 1:

This is a very well written manuscript describing results from a carefully conducted, thoughtfully discussed, and important study showing the dependency of postnatal development of dendritic organization of taste projection neurons in the nucleus of the solitary tract (NTS) on the presence of the α subunit of the epithelial sodium channel (αENaC) in taste bud cells. αENaC is a component of a passive sodium-specific ion channel that has been shown to be critical for the specific taste recognition of sodium (and lithium). The authors used genetically engineered mice that were lacking αENaC specifically in cytokeratin-19-expressing cells. All taste bud cells express this marker and so the gene deletion was largely specific to the peripheral gustatory system. Indeed, they confirmed in chorda tympani nerve recordings, that the combined signal arising from the response of anterior tongue taste buds to NaCl, in both adults and at postnatal day 20 (P20), was reduced and refractory to amiloride inhibition in the αENaC KOs. Thus, there is an absence of an amiloride-sensitive sodium signal, known to be critical sodium-specific taste-related behavior, throughout the developmental epoch examined in the αENaC KOs. The authors very reasonably conclude that the absence of this peripheral signal causes the NTS taste projection neurons to increase, rather than decrease, their a) dendritic field size and complexity, and b) chorda tympani terminal field size, as development proceeds past P20. This is important series of findings because it provides new details of activity-dependent development of gustatory circuits in rodent brain.

I have no major concerns. I think this is a very solid piece of work. However, I do have a set of recommendations for the authors to consider.

MAJOR: None.

MINOR:

1. Line 118. I recommend changing this word. Although it is commonly used by taste scientists, from a sensory point of view, I think it is misapplied. For me, “modality” refers to sensory systems (e.g., taste, smell, vision, etc.). I think it would be appropriate to change “single taste modality” to “a single receptor that is essential for sodium taste recognition”. I would change the use of the term “modality” throughout the entire manuscript.

2. Lines 132- 134. I will defer to the authors’ expertise, but couldn’t the effects of the KO manipulation on dendritic organization, in some ways, be considered dendritic development? Perhaps some rephrasing is in order.

3. Statistics. The methods are a little unclear about the statistical analysis involving the dendrite measures. It appears that all the neurons across animals were collapsed in a single group and treated as independent observations. I will not contest that because it is a common practice in neuroanatomy and neurophysiological research. More to the point, I find the data more than convincing and, in a way, the statistics are just a means to promote confidence. However, I recommend that the authors consider adding some information and analyses to the manuscript to allay any concerns.

• First, the methods should clearly state that all the neurons in a single group were collapsed.

• Second, the cells from individual animals within a group should be tested in an ANOVA. This should show that there are no fundamental differences across animals within a group lending some support for collapsing the data. In addition, the number of cells from each animal contributing to the analysis should be provided by group in a table or figure. The point here is to show that all the data are not particularly biased toward one or two animals in a group contributing a majority of the data.

• Third, in the analysis of the CT responses, the sample sizes are particularly low and I would suggest adding symbols for individual animal data overlaid on the means. This way, readers can see the spread and overlap between the distributions.

4. Figure 3. What does the line and asterisk above the graphs in 3b, c, and d mean? The caption merely says that *<.05. The type of comparisons to which this refers is unclear.

5. Line 550-551. The phrase “...one of the major pathways for ascending gustatory information” in referring to the solitary tract is awkward. It might be better to refer to this as, “...the source of all gustatory input from the periphery.”.

6. Line 641. It would be good to add: Ye Q, Heck GL, DeSimone JA (1993) Voltage dependence of the rat chorda tympani response to Na+ salts: Implications for the functional organization of taste receptor cells. J Neurophysiol 70:167-178.

7. Line 711. Chandrashekar et al (2010) did not test need-free detection of sodium. Given that amiloride strikingly increases detection threshold by an order of magnitude in both rats and mice, it is quite likely if the αENaC KOs were psychophysically tested for need free NaCl detection, they would display severely decreased sensitivity to the salt (see Geran LC, Spector AC (2000) Sodium taste detectability in rats is independent of anion size: the psychophysical characteristics of the transcellular sodium taste transduction pathway. Behav Neurosci 114:1229-1238. Also: Eylam S, Spector AC (2002) The effect of amiloride on operantly conditioned performance in an NaCl taste detection task and NaCl preference in C57BL/6J mice. Behav Neurosci 116:149-159.)

8. Line 711. Nor did Chandrashekar et al (2010) test aversion per se, but rather avoidance (see Schier LA, Spector AC (2019) The Functional and Neurobiological Properties of Bad Taste. Physiol Rev 99:605-663 for a discussion about the distinction).

Reviewer 2:

This is a straightforward, clear paper describing neuroanatomical and neurophysiological techniques to study the stimulus-dependency of development of dendritic fields of relay cells in the NST. The findings are Interesting and novel. The following concerns were noted:

Lines 103-106 - statement is a little confusing. Do other tastes not increase in magnitude during development? It is certainly interesting that lack of experience with a single type of taste modality is enough to disrupt developmental pruning in the taste system, although one could argue that these mice have limited exposure to other tastants/stimuli as well. I would like to see some discussion around this point, including whether other experiments have found similar central changes depending on exposure to other taste qualities. Sweet taste would be an interesting one, and the responses of NST neurons in sweet-KO mice have been investigated (e.g. Lemon and Margolskee, 2009).

It feels like the sample sizes are somewhat small for successful use of parametric statistics, especially for the physiology experiments where comparisons are made between 3,2,5 and 3 mice in each group. Did the authors conduct assumption tests prior to ANOVAs?

In Figure 2, it’s hard to distinguish the individual circles, especially between red and pink. The authors should consider expanding the size of the portion of the schematic that actually contains the cells, and eliminating much of the empty space.

Line 436-440: Is the comparison here supposed to be P20 ENaC KO mice vs. adult ENaC KO mice?

Figure 3, and Experimental Design and Statistical Analyses: The neuroanatomical data that are analyzed are pooled data from all mice within a particular treatment group. However, this doesn’t give any sense as to whether data are consistent across individual mice within a treatment group. Was this factor analyzed? Was there significant variation in number of labeled cells per mouse within groups? This should be commented on, at least in in the statistical analysis section.

Line 620: “In adult aENaC KO mice, increasing the concentration of NaCl did not result in statistical increases in CT responses”. What is the statistic here? In a 2- or 3-way ANOVA, with concentration as a repeated measures factor, it certainly looks as though there would be a significant effect of concentration.

## Author Response

We thank the two reviewers for their comments regarding our manuscript. We made a number of changes including additional analyses and text in response to many of the concerns raised by the reviewers. These changes, along with the reviewer’s comments (shown in bold test), are outlined below. Text excerpts from our manuscript are shown in insets and in italics, and new text resulting from the reviewer’s comments are highlighted in red font.

Reviewer Comments:

Reviewer #1:

Major Concerns: None

Minor Concerns:

1. Line 118. I recommend changing this word. Although it is commonly used by taste scientists, from a sensory point of view, I think it is misapplied. For me, “modality” refers to sensory systems (e.g., taste, smell, vision, etc.). I think it would be appropriate to change “single taste modality” to “a single receptor that is essential for sodium taste recognition”. I would change the use of the term “modality” throughout the entire manuscript.

We thank the reviewer for this observation as the term “modality” does tend to imply whole sensory systems rather than specific aspects within a given sensory system. We took the reviewers point to heart and replaced the term “modality” throughout the manuscript. One example of this change can be seen at Line 119, where the reviewer references.

Given the large effects that removing sodium salt taste has on gustatory terminal fields, one might expect removing ENaC-mediated neural activity would also affect their postsynaptic targets that send afferent taste information to more central structures.

2. Lines 132- 134. I will defer to the authors’ expertise, but couldn’t the effects of the KO manipulation on dendritic organization, in some ways, be considered dendritic development? Perhaps some rephrasing is in order.

We thank the reviewer for this point as they are correct in stating that the effects of the KO manipulation could be considered a form of dendritic development on their own. The intention of the statement referenced by the reviewer was to illustrate that ENaC-mediated activity was not required for the dendritic development seen in the control population. We have altered the text to make this statement more clearly about the normal development seen in control mice.

Unexpectedly, we found that the size of dendritic fields in ENaC KO mice became larger and more complex than controls with age, while the general shape of the dendritic fields remained similar to controls. Our surprising findings indicate that, while ENaC-mediated sodium salt taste activity appears not to be involved in the normal dendritic development of NST relay cells in control mice, it is required to maintain their mature dendritic organization.

3. Statistics. The methods are a little unclear about the statistical analysis involving the dendrite measures. It appears that all the neurons across animals were collapsed in a single group and treated as independent observations. I will not contest that because it is a common practice in neuroanatomy and neurophysiological research. More to the point, I find the data more than convincing and, in a way, the statistics are just a means to promote confidence. However, I recommend that the authors consider adding some information and analyses to the manuscript to allay any concerns.

• First, the methods should clearly state that all the neurons in a single group were collapsed.

We thank the reviewer for the feedback on our statistical analysis regarding the dendritic measures. Indeed, we collapsed all the cells of a given group into a single larger group for analysis. We have updated our methods (under “Experimental Design and Statistical Analyses” section) to include this important point.

Experiments investigating NST relay cell dendritic architecture were conducted in control and αENaC KO mice at P18-22 (P20 Control, 4 mice, 2 male/2 female; P20 αENaC KO, 3 mice, 2 male/1 female) and P60-120 (Adult Control, 8 mice, 3 male/5 female; Adult αENaC KO, 10 mice, 4 male/6 female). Neurons collected from different mice within the same age X experimental group were collapsed into a single group, resulting in four larger neural populations (22 cells from P20 control mice, 31 cells from P20 αENaC KO mice, 123 cells from adult control mice, 81 cells from adult αENaC KO mice).

• Second, the cells from individual animals within a group should be tested in an ANOVA. This should show that there are no fundamental differences across animals within a group lending some support for collapsing the data. In addition, the number of cells from each animal contributing to the analysis should be provided by group in a table or figure. The point here is to show that all the data are not particularly biased toward one or two animals in a group contributing a majority of the data.

Again, we thank the reviewer for suggesting further analyses to support our method of combining cells across animals within a given group. We appreciate this point and have created two Extended Data Figures to alleviate these concerns. In the first of these new figures, we report the number of labeled cells that we were able to collect from all of the animals used in this study (Extended Data Fig. 3-1a). In this figure we also calculate the average number of dendritic branch points, average dendritic length and average dendritic complexity for each animal in the four groups (Extended Data Fig. 3-1b,c,d). We believe that this illustrates that while there is some variability across animals within a given group, the differences we report are largely due to wholesale changes across the multiple animals from which we record rather than due to one or two animals. To further illustrate this point, we created a second Extended Data Figure (Extended Data Fig. 3-2) where we plot the dendritic complexity measure of each of our labeled neurons as a function of the animal from which they come from. We chose to illustrate this point with our dendritic complexity data as we believe that the changes in dendritic complexity we report are the most important of all the dendritic measures. This is because dendritic complexity is a measure that takes into account multiple aspects of the dendritic architecture (branch tip order, number of dendritic endings, dendritic length and number of primary dendrites). This figure illustrates two main points. First, that there is variability in this measurement that is not captured by solely reporting the mean of each animal (or each collapsed group, to the reviewer’s point). More importantly however, it illustrates that despite the variability within a single animal, the data is largely similar across animals of a given group, providing credence for collapsing data across animals into a single group. We do note that the dendritic complexity measures from one or two of the adult control mice (the first and fourth control mouse in Extended Data Fig. 3- 2c) were substantially higher than what we see in the other adult control mice. However, given the direction of this difference, these animals are actually working against the general trend that we report in the main text rather than driving it. Finally, we have added to the text to include a brief discussion of these figures and the important concerns raised by both reviewers.

There was variability in the number of labeled cells that we were able to collect from each mouse, especially within the adult control group (Extended Data Fig. 3-1a). Despite this variability, a one-way ANOVA did not find a significant difference in the average number of cells labeled between groups (Extended Data Fig. 3-1a, F(3,24) = 2.849, p = 0.062). We do note that this statistical test was near but not at the threshold for statistical significance.

The mean number of dendritic branch points of relay cells from P20 control, adult control and P20 αENaC KO mice were not significantly different from each other (Fig 3B, p > 0.05). Therefore, the variability in the mean number of dendritic branch points of all neurons among individual mice is not responsible for the significant group-related differences we report for dendritic branch points (Extended Data Fig 3-1B).

The mean dendritic length in relay cells in P20 control, adult control and P20 αENaC KO mice were not different from each other (Fig 3C, p > 0.05). Calculating the mean dendritic length of all neurons from any one mouse illustrates that, while there is some variability from animal to animal, the significant differences we report are not due solely to one or two animals (Extended Data Fig. 3-1C).

A large majority of relay cells from adult ENaC KO mice (81.5%) had a DCI larger than the median DCI of the adult control population. Calculating the mean dendritic complexity of all neurons from any one mouse shows that while there is variability from animal to animal, the significant differences we report are not driven by a few outliers (Extended Data Fig. 3-1D, Extended Data Fig.3-2).

• Third, in the analysis of the CT responses, the sample sizes are particularly low and I would suggest adding symbols for individual animal data overlaid on the means. This way, readers can see the spread and overlap between the distributions.

We greatly appreciate this suggestion and have replaced the error bars in our CT responses figure (Figure 6) with the individual animal data points along with the means, represented by the solid and dotted lines. Furthermore, we have edited the caption to reflect this change.

E, Mean Relative taste responses from P20 control mice (light blue) and adult control mice (dark blue) to a concentration series of NaCl before (solid lines) and after (dotted lines) lingual application of amiloride. Responses from individual animals before amiloride are represented by squares while responses after amiloride are represented with triangles. F, Mean relative taste responses from P20 ENaC KO mice (light red) and adult ENaC KO mice (dark red) to a concentration series of NaCl before (solid lines) and after (dotted lines) lingual application of amiloride. Responses from individual animals before amiloride are represented by squares while responses after amiloride are represented with triangles. * p < 0.05

4. Figure 3. What does the line and asterisk above the graphs in 3b, c, and d mean? The caption merely says that *<.05. The type of comparisons to which this refers is unclear.

We appreciate that the reviewer notes that our attempt to illustrate statistically significance differences in Figure 3 are not clear enough. To more clearly illustrate these differences, we have done two things. First, we altered the original line and asterisk in Figure 3 to a more common bracket that clearly illustrates which groups are significantly different from each other. Second, we altered the caption for figure 3 to clearly articulate the statistical comparisons that are being done. Below are the specific changes we made to Figure 3’s caption.

D, Mean (± SEM) dendritic complexity (see methods for details) of relay cells from P20 control (light blue), adult control (dark blue), P20 ENaC KO (light red), and adult ENaC KO (dark red) mice. Lines and brackets above B, C and D denote comparisons that reached statistical significance using a one-way ANOVA while correcting for multiple comparisons * < p 0.05.

5. Line 550-551. The phrase “...one of the major pathways for ascending gustatory information” in referring to the solitary tract is awkward. It might be better to refer to this as, “...the source of all gustatory input from the periphery.”.

We agree with the reviewer’s suggestion to alter the text referring to the solitary tract to be more definitive about its important role in carrying taste-related information from the periphery. We have made the following change to the text per the reviewer’s suggestion.

Typically, these relay cells orient their dendritic fields parallel to the solitary tract, the source of gustatory input from the periphery (Davis, 1988; Corson and Erisir, 2013).

6. Line 641. It would be good to add: Ye Q, Heck GL, DeSimone JA (1993) Voltage dependence of the rat chorda tympani response to Na+ salts: Implications for the functional organization of taste receptor cells. J Neurophysiol 70:167-178.

We appreciate the reviewer’s suggestion to add the citation Ye et al, 1993 to this portion of our manuscript. Indeed, the findings of Ye et al (1993) are relevant to the statement that amiloride-insensitive salt responses (or amiloride-insensitive component (AIC) in Ye et al 1993) are dependent upon the anion portion of salts. Furthermore, Ye et al (1993) studied this effect in the chorda tympani, making the results directly relevant to the findings of this study. We have added this citation to our study and thank the reviewer for the suggestion.

Multiple studies have shown these amiloride-insensitive salt responses are dependent upon the anion component of salts (chloride here) (Formaker and Hill, 1988; Elliot and Simon, 1990; Ye et al., 1993; Lewandowski et al., 2016; Roebber et al., 2019).

7. Line 711. Chandrashekar et al (2010) did not test need-free detection of sodium. Given that amiloride strikingly increases detection threshold by an order of magnitude in both rats and mice, it is quite likely if the αENaC KOs were psychophysically tested for need free NaCl detection, they would display severely decreased sensitivity to the salt (see Geran LC, Spector AC (2000) Sodium taste detectability in rats is independent of anion size: the psychophysical characteristics of the transcellular sodium taste transduction pathway. Behav Neurosci 114:1229-1238. Also: Eylam S, Spector AC (2002) The effect of amiloride on operantly conditioned performance in an NaCl taste detection task and NaCl preference in C57BL/6J mice. Behav Neurosci 116:149-159.)

We thank the reviewer for bringing up these important distinctions. Our statement that Chandrashekar et al (2010) tested the need-free detection of sodium was in fact as the incorrect and we appreciate the clarification on this point. The reviewer’s prediction on the psychophysical responses of αENaC KO mice are interesting and would be a very interesting follow-up to this current work. To more accurately reflect the findings of the work of Chandrashekar et al. (2010) we have made the follow changes to our manuscript.

For example, Chandrashekar et al. (2010) report that taste-related behaviors are largely the same between ENaC KO and control mice, with the exception of responses to appetitive concentrations of NaCl. Specifically, it appears as though appetitive aspects of NaCl in salt appetite are blunted or eliminated in these mice, the normal avoidance of high concentrations of NaCl are left intact. Moreover, our findings here suggest a significant alteration in the way that brainstem taste neurons will process (i.e., code) taste stimuli.

8. Line 711. Nor did Chandrashekar et al (2010) test aversion per se, but rather avoidance (see Schier LA, Spector AC (2019) The Functional and Neurobiological Properties of Bad Taste. Physiol Rev 99:605-663 for a discussion about the distinction).

Again, we appreciate the clarification on the findings of Chandrashekar et al (2010). To alleviate the important concerns raised by the reviewer we have altered to text to accurately reflect the findings of Chandrashekar et al (2010). The specific changes can be seen above in our response to this reviewer’s 7th suggested edit.

Reviewer 2:

1. Lines 103-106 - statement is a little confusing. Do other tastes not increase in magnitude during development? It is certainly interesting that lack of experience with a single type of taste modality is enough to disrupt developmental pruning in the taste system, although one could argue that these mice have limited exposure to other tastants/stimuli as well. I would like to see some discussion around this point, including whether other experiments have found similar central changes depending on exposure to other taste qualities. Sweet taste would be an interesting one, and the responses of NST neurons in sweet-KO mice have been investigated (e.g. Lemon and Margolskee, 2009).

We appreciate the reviewer’s points regarding the ‘normal’ development of taste circuits in the NST and how dependent this development is on salt taste alone. While salt-taste activity undergoes the largest developmental increase in control animals, sweet taste also undergoes a similar, albeit lesser, developmental increase. Thus, the reviewer is correct in stating that sweet taste would may be an interesting target for additional studies investigating the relationship between the anatomical development of taste circuits and developing peripheral input. Furthermore, the reviewer is also correct in stating that there is a fair argument to be made that our control animals are actually just slightly less deprived than our ENaC KO mice. The ‘normal’ development that we describe is most likely very specifically related to the limited environment in which mice are typically housed. We had considered using an environmental enrichment paradigm to increase access to taste-input in an experimental group of mice to attempt to further investigate the relationship between peripheral taste input and NST development, but the conclusions that may be drawn from these studies are limited as it is hard to rule out post-ingestional effects as a factor in the results. Regardless, the effects we show are specifically related to differences in sodium-taste input as the gustatory environment that was available to both groups of mice were the same, the only difference being our experimental mice lacked the transduction machinery needed for salty-taste. We appreciate the reviewer’s point and therefore have altered the text to also briefly mention that sweet-taste responses undergo a similar postnatal development.

This anatomical development occurs in tandem with a nearly two-fold developmental increase in the magnitude of salty-taste responses (and to a lesser extent sweet-taste responses) in the CT (Yamada, 1980; Hill and Bour, 1985; Hill, 1988; Zheng et al., 2014), suggesting increases in peripheral input regulates terminal field “pruning”.

We have also edited the text to more accurately reflect that salt-taste activity is important for the normal development in our control mice. See an example below.

Therefore, salt taste activity from a single transduction pathway is necessary for the proper development and for the maintenance of gustatory terminal fields in control mice.

2. It feels like the sample sizes are somewhat small for successful use of parametric statistics, especially for the physiology experiments where comparisons are made between 3,2,5 and 3 mice in each group. Did the authors conduct assumption tests prior to ANOVAs?

We appreciate the reviewers point regarding the low sample sizes in our physiology experiments. Indeed, our physiological experiments are hindered by low sample sizes, making statistical comparisons difficult. Given that previous publications from our lab (Skyberg et al., 2017 and Sun et al. 2017) and other labs (Chandrashekar et al., 2010) report similar physiological effects from genetic removal of ENaCs we are confident in our results despite the low sample sizes. We addressed this issue to by doing two additional statistical tests, both with a focus on taste responses to 0.25M and 0.5M NaCl. The first test, which is what we now present in the paper, are multiple-t tests with the Holm-Sidak correction of the alpha level. We also did the Mann-Whitney non parametric tests. Results from both tests showed the same statistical significant differences, which were also similar to our original ANOVA tests. We added this statistical design to the text, as can be seen below.

The relative responses to 0.25M and 0.5M NaCl in P20 control mice were significantly less (40-50%) than the respective responses in adult control mice (Fig 6E; t(6) = 2.9 - 4.9; significant posttest, p = 0.03 and 0.005 for 0.25M NaCl and 0.5M NaCl, respectively). Interestingly, there were also differences in NaCl responses after lingual application of amiloride between P20 control and adult control mice. Relative CT responses to 0.5M NaCl after amiloride from P20 control mice were 50% less than the respective responses in adult control mice after amiloride (Fig 6E; t(6) = 4.2; p = 0.01).

By comparison, conditionally deleting the Scnn1a gene from taste bud cells had selective effects on CT whole nerve taste responses. Like adult control mice, increasing the concentration of NaCl in adult αENaC KO mice resulted in increased CT responses; however, the relative magnitude of these responses were lower than that of adult control mice at all stimulus concentrations (Fig 6E-F). However, the only group-related difference occurred for taste responses to 0.5M NaCl (t(6) = 15.0; p = 0.001) and is similar to that previously reported (Chandrashekar et al., 2010; Skyberg et al., 2017; Sun et al., 2017).

Also, we updated our Experimental Design and Statistical Analyses section in our Methods to reflect this additional statistical test.

Experiments examining chorda tympani whole nerve taste responses were done in control and αENaC KO mice at P20 (P20 Control, 3 mice, 2 male/1 female; P20 αENaC KO, 2 mice, 1 male/1 female) and P60-120 (Adult Control, 5 mice, 3 male/2 female; Adult αENaC KO, 3 mice, 1 male/2 female). Due to the small number of observations in each group, we did two statistical tests that are most appropriate for these types of comparisons. For both tests, we focused on group- and age-related differences at 0.25M NaCl and 0.5M NaCl. Taste responses to lower concentrations showed no differences between control and αENaC KO mice at either age. First, we compared mean (± SEM) relative CT responses to 0.25M and 0.5M NaCl (normalized to 0.5M NH4Cl responses) between P20 control and P20 ENaC KO mice and between adult control and adult ENaC KO mice with multiple t-tests, and with the alpha level (0.05) corrected by the Holm-Sidak correction method. We also did the Mann-Whitney nonparametric test for the same comparisons. Both statistical methods provided the same outcome. We present only the statistical findings from the multiple t-test comparisons.

3. In Figure 2, it’s hard to distinguish the individual circles, especially between red and pink. The authors should consider expanding the size of the portion of the schematic that actually contains the cells, and eliminating much of the empty space.

We agree with the reviewer that including traces of the whole brainstem, while somewhat useful, ultimately makes it hard to distinguish the individual points within the NST. Therefore, we have taken the reviewers suggestion and remade figure 2 by removing much of the empty (non-NST) space. While this means that we must remove some of the important landmarks we use to identify which portion of the dorsal-ventral axis the brain section comes from, we do discuss these landmarks in detail in the text. Furthermore, removing these aspects of the figure help to highlight the most important points of the figure, as the reviewer point out. We have also changed the figure caption to reflect these changes.

Figure 2. Relay cell locations throughout the dorsal ventral axis of the NST. The location of each cell from P20 control (light blue), adult control (dark blue), P20 ENaC KO (light red), and adult ENaC KO (dark red) mice are projected onto representative tracings of the horizontally-sectioned NST. The far dorsal, dorsal, intermediate, and ventral NST were located 100-300mm, 300-500mm, 500-800mm and 800-1200mm from the surface of the brainstem, respectively. Scale bar, 500mm; NST, nucleus of the solitary tract; st, solitary tract; R, rostral; L, lateral.

4. Line 436-440: Is the comparison here supposed to be P20 ENaC KO mice vs. adult ENaC KO mice?

We thank the reviewer for noticing this. It was in fact a typo and was supposed to be a comparison between P20 and adult ENaC KO mice as the reviewer noted. We have changed to text to reflect that as can be seen below.

Finally, the dorsal-ventral distribution of P20 ENaC KO relay cells was not significantly different than that of the adult ENaC KO population (X2 (3) = 3.24, p=0.356)

5. Figure 3, and Experimental Design and Statistical Analyses: The neuroanatomical data that are analyzed are pooled data from all mice within a particular treatment group. However, this doesn’t give any sense as to whether data are consistent across individual mice within a treatment group. Was this factor analyzed? Was there significant variation in number of labeled cells per mouse within groups? This should be commented on, at least in in the statistical analysis section.

We greatly thank the reviewer for raising this concern. In fact, both reviewers brought up similar points surround these issues. Indeed, the findings from our study would be greatly bolstered by analyses showing that one or two mice are not solely responsible for driving the effects we see after collapsing all cells into larger experimental and control groups. We have done a number of things to alleviate these concerns. First, as reviewer one noted, we now clearly mention that we combine neurons from animals of the same condition into a single, larger group. Second, and more directly relevant to this reviewers’ concerns, we have added two Extended Data figures. The first of these figures includes a plot (Extended Data Fig. 3-1a) showing the number of cells labeled from every mouse used in this study. While there is variability in the number of labeled cells from each animal, particularly in the adult control group, an ANOVA failed to show that these differences were statistically significant. We have added some text to our results to address this This figure also includes plots showing the average number of dendritic branches (Extended Data Fig. 3-1b), average dendritic length (Extended Data Fig. 3-1c), and average dendritic complexity (Extended Data Fig. 3-1d) of each of the mice used in this study. To be specific, for each of these measurements we averaged across all the labeled neurons that came from an individual mouse. This figure shows that while there is variability across animals within a single condition (often due to variability in the number of labeled cells), the larger effects that we report in the manuscript are not due to one or two animals that are wildly different than the rest. To further illustrate this point, we also created a second Extended Data figure where we plot the dendritic complexity measure of every cell used in this study as a function of the animal from which it came (Extended Data Fig. 3-2). We chose to illustrate this point with our dendritic complexity data as we believe that the changes in dendritic complexity that we report are the most important of all the dendritic measures. This is because dendritic complexity is a measure that takes into account multiple aspects of the dendritic architecture (branch tip order, number of dendritic endings, dendritic length and number of primary dendrites). This figure illustrates two main points. First, that there is variability in this measurement that is not captured by solely reporting the mean of each animal (or each collapsed group, to the reviewer’s point). More importantly however, it illustrates that despite the variability within a single animal, the data is similar across animals of a given group, providing credence for collapsing data across animals into a single group. We do note that the dendritic complexity measures from one or two of the adult control mice (the first and fourth control mouse in Extended Data Fig. 3-2c) were substantially higher than what we see in the other adult control mice. However, given the direction of this difference, these animals are actually working against the general trend that we report in the main text rather than driving it. Finally, we have added to the text to include a brief discussion of these figures along with statistical comparisons to show that not one mouse is driving the effects we originally report in the manuscript. We believe these alterations to our manuscript will alleviate the concerns of both reviewers and we thank the reviewers for making these important suggestions.

Lines 421-426:There was variability in the number of labeled cells that we were able to collect from each mouse, especially within the adult control group (Extended Data Fig. 3-1A). Despite this variability, a one-way ANOVA did not find a significant difference in the average number of cells labeled between groups (Extended Data Fig. 3-1A, F(3,24) = 2.849, p = 0.062). We note that this statistical test was near but not at the threshold for statistical significance.

Lines 470-475:The mean number of dendritic branch points of relay cells from P20 control, adult control and P20 αENaC KO mice were not significantly different from each other (Fig 3B, p > 0.05). Therefore, the variability in the mean number of dendritic branch points of all neurons among individual mice is not responsible for the significant group-related differences we report for dendritic branch points (Extended Data Fig. 3-1B).

Lines 481-486:The mean dendritic length in relay cells in P20 control, adult control and P20 αENaC KO mice were not different from each other (Fig 3C, p > 0.05). Calculating the mean dendritic length of all neurons from any one mouse illustrates that, while there is some variability from animal to animal, the significant differences we report are not due solely to one or two animals (Extended Data Fig. 3-1C).

Lines 499-503:A large majority of relay cells from adult ENaC KO mice (81.5%) had a DCI larger than the median DCI of the adult control population. Calculating the mean dendritic complexity of all neurons from any one mouse shows that while there is variability from animal to animal, the significant differences we report are not driven by a few outliers (Extended Data Fig. 3-1D, Extended Data Fig. 3-2).

6. Line 620: “In adult aENaC KO mice, increasing the concentration of NaCl did not result in statistical increases in CT responses”. What is the statistic here? In a 2- or 3-way ANOVA, with concentration as a repeated measures factor, it certainly looks as though there would be a significant effect of concentration.

We agree with the reviewer that data certainly does not seem to support the comment that “there is not a statistically significant increase in the responses from adult ENaC KO mice”. That comment was incorrect, we appreciate the reviewer’s astute observation and apologize for this oversight. In the end, we decided to remove the statement altogether for a couple reasons. First, we did not make a similar statistical comparison in any of the other three groups (most importantly the adult control group). Second, the two major points of this section of the text are not dependent upon that statistical comparison at all. Those main points are that adult ENaC KO mice have significantly diminished responses when compared to their adult control counterparts and that these adult ENaC KO responses are almost entirely amiloride-insensitive, suggesting that the epithelial sodium channel is mediating these adult ENaC KO responses. We have made appropriate changes to the text to reflect the decision to remove the statement of concern.

By comparison, conditionally deleting the Scnn1a gene from taste bud cells had selective effects on CT whole nerve taste responses. Like adult control mice, increasing the concentration of NaCl in adult αENaC KO mice resulted in increased CT responses; however, the relative magnitude of these responses were lower than that of adult control mice at all stimulus concentrations (Fig 6E-F). However, the only group-related difference occurred for taste responses to 0.5M NaCl (t(6) = 15.0; p = 0.001) and is similar to that previously reported (Chandrashekar et al., 2010; Skyberg et al., 2017; Sun et al., 2017).
