# Supplementary figures and images for: Selective Removal of Sodium Salt Taste Disrupts the Maintenance of Dendritic Architecture of Gustatory Relay Neurons in the Mouse Nucleus of the Solitary Tract
Source: eNeuro. 2020 Oct 19;7(5):ENEURO.0140-20.2020. doi: 10.1523/ENEURO.0140-20.2020 (PMC7598914; doi:10.1523/ENEURO.0140-20.2020)

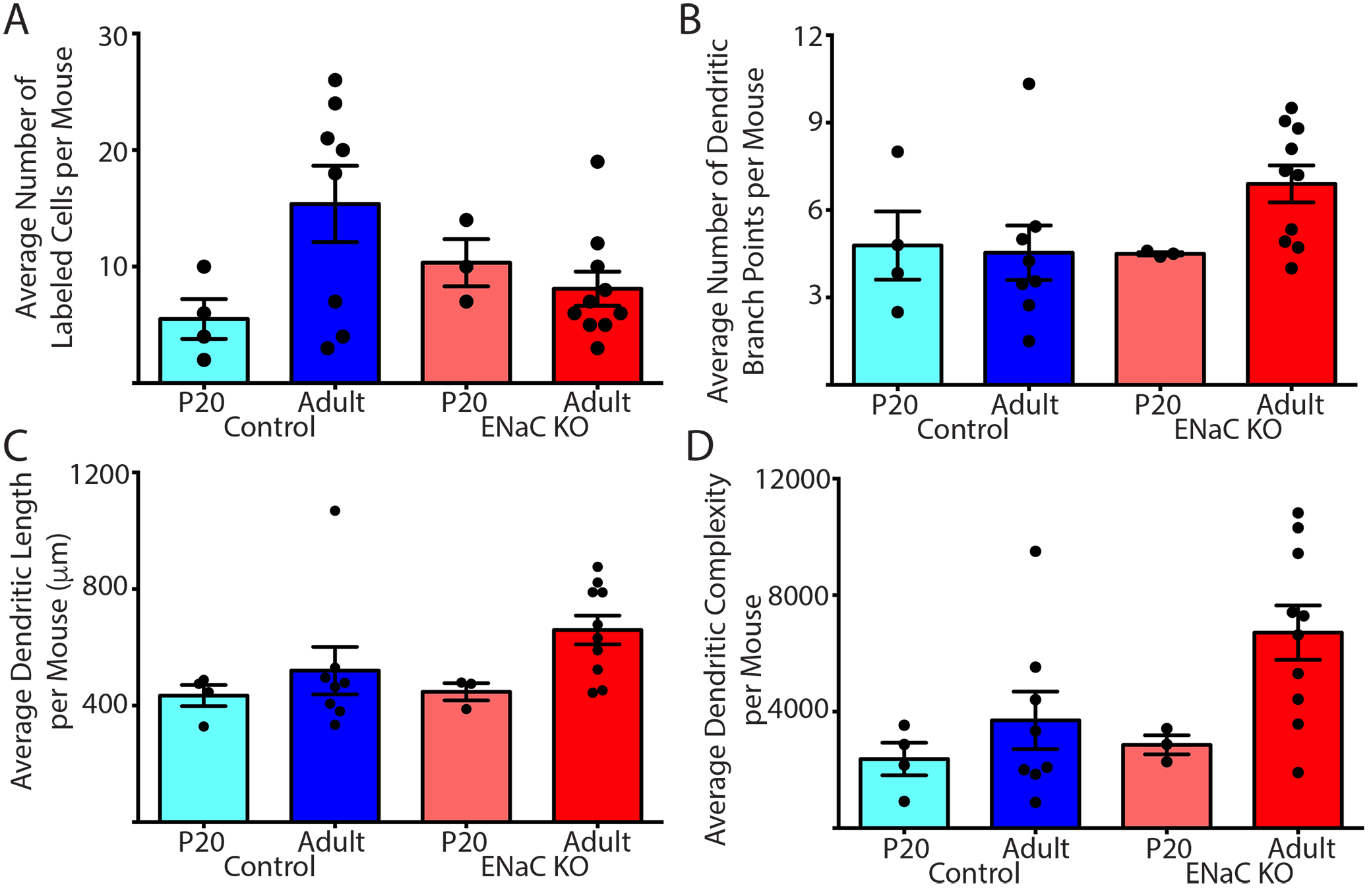

Supplement: Extended Data Figure 3-1 — Average dendritic measurements for each animal. A, Number of NST relay cells labeled in each animal used in this study. Each dot refers to the number of cells labeled in one animal while the bars indicate the group means (± SEM). B, Average number of dendritic branch points occurring in neurons from one animal. Each dot reflects the average of each mouse while the bars indicate the group means (± SEM). C, Same as B but for average dendritic length. D, Same as B but for average dendritic complexity. Download Figure 3-1, TIF file. [file enu-eN-NWR-0140-20-s01.tif]

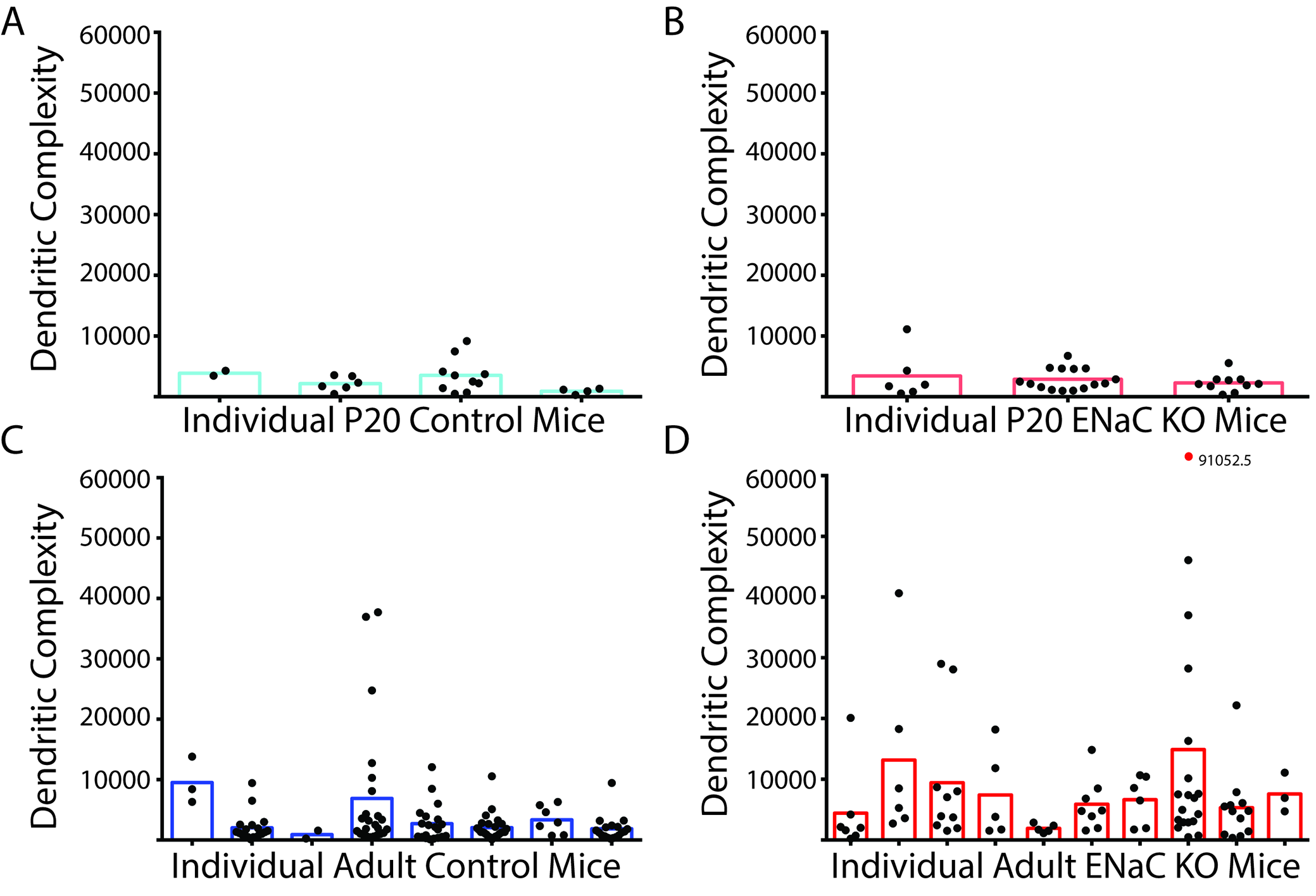

Supplement: Extended Data Figure 3-2 — Dendritic complexity for every cell used in this study organized by mouse from which it came. A, Dendritic complexity of all neurons from the 4 P20 control mice. B, Dendritic complexity of all neurons from the 3 P20 ENaC KO mice. C, Dendritic complexity of all neurons from the 8 adult control mice. D, Dendritic complexity of all neurons from the 10 adult ENaC KO mice. Dots indicate dendritic complexity of a single cell while the bars indicate the mean dendritic complexity of all the cells from one animal. The dendritic complexity of one adult ENaC KO cell fell outside of the bounds of the y axis. This cell is represented by a red dot and its dendritic complexity measure is to the right of the red dot. Download Figure 3-2, TIF file. [file enu-eN-NWR-0140-20-s03.tif]
